# Super-resolution microscopy reveals photoreceptor-specific subciliary location and function of ciliopathy-associated protein CEP290

**DOI:** 10.1172/jci.insight.145256

**Published:** 2021-10-22

**Authors:** Valencia L. Potter, Abigail R. Moye, Michael A. Robichaux, Theodore G. Wensel

**Affiliations:** 1 Verna and Marrs McLean Department of Biochemistry and Molecular Biology,; 2 Program in Developmental Biology, Graduate School of Biomedical Sciences, and; 3 Medical Scientist Training Program, Baylor College of Medicine, Houston, Texas, USA.; 4 Departments of Ophthalmology and Biochemistry, West Virginia University School of Medicine, Morgantown, West Virginia, USA.

**Keywords:** Cell Biology, Ophthalmology, Genetic diseases, Mouse models, Retinopathy

## Abstract

Mutations in the cilium-associated protein CEP290 cause retinal degeneration as part of multiorgan ciliopathies or as retina-specific diseases. The precise location and the functional roles of CEP290 within cilia and, specifically, the connecting cilia (CC) of photoreceptors, remain unclear. We used super-resolution fluorescence microscopy and electron microscopy to localize CEP290 in the CC and in the primary cilia of cultured cells with subdiffraction resolution and to determine effects of CEP290 deficiency in 3 mutant models. Radially, CEP290 localizes in close proximity to the microtubule doublets in the region between the doublets and the ciliary membrane. Longitudinally, it is distributed throughout the length of the CC whereas it is confined to the very base of primary cilia in human retinal pigment epithelium-1 cells. We found Y-shaped links, ciliary substructures between microtubules and membrane, throughout the length of the CC. Severe CEP290 deficiencies in mouse models did not prevent assembly of cilia or cause obvious mislocalization of ciliary components in early stages of degeneration. There were fewer cilia and no normal outer segments in the mutants, but the Y-shaped links were clearly present. These results point to photoreceptor-specific functions of CEP290 essential for CC maturation and stability following the earliest stages of ciliogenesis.

## Introduction

Ciliopathies, genetic defects in components of primary and motile cilia, lead to a host of diseases with diverse presentations, which reflect the diverse functions of cilia (1, 2). Primary cilia, nonmotile, hair-like protrusions found on virtually all mammalian cells, are elaborate structures with hundreds of protein constituents that function as cell antennae, collecting and relaying external stimuli to regulate cellular processes and maintain homeostasis. An interesting subset of ciliopathy genes can be associated either with multisyndromic disease, often including retinal degeneration, or with retina-specific disease (3), depending on the allele and possibly on the genetic background and other factors. Little is known about the factors that determine the precise manifestations of each ciliopathy or about the pathophysiological mechanisms involved in retinal degeneration. The occurrence of different ciliopathy phenotypes likely depends on the precise positioning of structural and functional components.

Defects in the gene encoding centrosomal protein of 290 kDa (*CEP290*) are the leading genetic cause of the severe blinding condition known as Leber congenital amaurosis (LCA) (4–7). In addition to LCA, different *CEP290* mutations can give rise to nonsyndromic retinitis pigmentosa (8) and multisyndromic disorders with associated retinal degeneration, such as Bardet-Biedl syndrome and Meckel-Gruber syndrome (5, 9–14). Understanding the functions of the protein CEP290, both in photoreceptor sensory cilia and in other primary cilia is critical to understanding these diseases, and to guiding therapeutic interventions and genetic counseling.

Primary cilia nucleate from a mother centriole at the apical surface of the cell and have a specialized region at their base called the transition zone, beyond which extends the ciliary axoneme, a bundle of 9 + 0 microtubule doublets. The transition zone ranges from approximately 200 to 300 nm in length and is approximately 300 nm in diameter, depending on cell type (1, 2, 15–18). Within the transition zone, there are filamentous structures extending from the outer surface of the axonemal microtubules to the ciliary membrane, often referred to as Y-shaped links or Y-links (the term used throughout here) because of their appearance in electron micrographs of ciliary cross sections stained with heavy metals (19, 20). Recent electron tomographic studies suggest that their true structures in 3 dimensions are not very Y-like but confirm that they are narrower at the microtubule end with wider bifurcations at the membrane (21, 22). The molecular composition of these structures is unknown. The ciliary membrane, while continuous with the plasma membrane, contains a unique set of membrane proteins, such as ion channels and receptors (15, 23, 24). An elaborate but poorly understood network of molecular machinery within the primary cilium is necessary to ensure proper transport and distribution of ciliary components and to prevent misaccumulation of nonciliary proteins.

**Figure 1:**
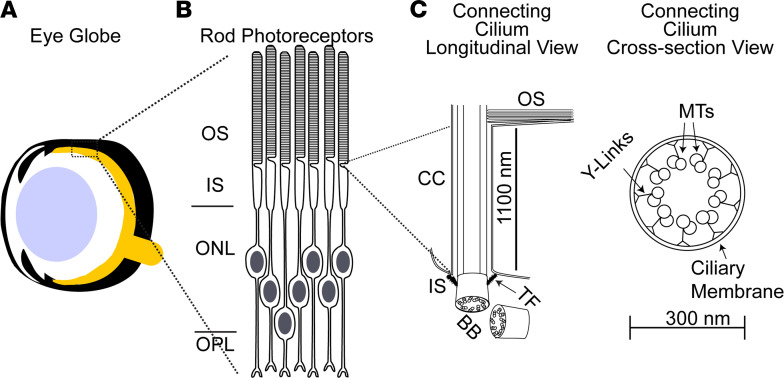
The photoreceptor CC. (**A**) The photoreceptors are the most posterior cells in the neural retina at the back of the eye. (**B**) Rod photoreceptor cells are distributed across 4 layers of the retina. (**C**) The CC links the outer segment to the inner segment. The dashed link shows the portion of the CC in the cross-sectional view. The dimensions of the CC are approximately 1100 nm by 300 nm. OS, outer segment; IS, inner segment; ONL, outer nuclear layer; OPL, outer plexiform layer; CC, connecting cilium; BB, basal body; TF, transition fibers; MT, microtubule doublets.

Among the numerous cell types that rely on primary cilia for proper functioning, rod and cone photoreceptor cells of the vertebrate retina carry out their entire phototransduction cascade within their cilia, making cilia essential for vision (reviewed in ref. 25). The modified primary cilia of photoreceptors contain a light-sensing outer segment and the connecting cilium (CC), which connects the outer and inner segments. The axoneme extends from the CC into the outer segment, with microtubules extending first as doublets, and then as singlets. The light-sensing disc membranes are anchored to the axonemal microtubules. Since proteins are synthesized in the inner segment of photoreceptor cells, the CC is an essential region for the trafficking of phototransduction proteins to the outer segment. The mouse CC is approximately 1100 nm in length and approximately 300 nm in diameter (Figure 1) (26). Inside the ring of 9 microtubule doublets is the lumen, containing calcium-binding proteins called centrins (27–31), and extending from the doublets to the ciliary membrane are the Y-links (Figure 1C), which have been proposed to aid in maintaining the structural integrity of the CC (32). The distribution of Y-links along the length of the CC has not been unequivocally determined; in most ciliated cells they are restricted to the basal region of the cilia as densities that connect the axoneme and membrane, often lacking strict 9-fold symmetry. However, connections have been observed throughout the length of some primary cilia (21). The structures of the Y-links in the CC and other mammalian primary cilia appear similar, but in no cell type are their molecular components known.

CEP290 has been localized to rod CC, and based on studies in vitro and in motile cilia (e.g., *Chlamydomonas* flagella, where it is also known as POC3), it has been proposed to play important roles in ciliogenesis, protein recruitment to the centriolar satellites, structural support of the cilium, and regulation of ciliary protein trafficking (33–35). The function of CEP290 in photoreceptors, however, remains uncertain. It has recently been proposed that retina-specific ciliopathies can arise from defects in retina-specific structures or aberrations in spatial distributions of components within the sensory cilia (outer segments plus CC) of rods and cones. In a recent study using methods similar to those employed here, we observed that a number of ciliary components, including CEP290, undergo photoreceptor-specific redistribution in the absence of the product of another LCA-associated gene, spermatogenesis associated 7 (*Spata7*) (36). These results and the spectrum of CEP290-associated disease suggest there may be functions of CEP290 that are specific to retinal photoreceptor cilia.

A limitation of most previous studies examining localization of CEP290 in photoreceptors has been the resolution limit of conventional light microscopy. Given that the entire width of the CC is approximately 300 nm, only slightly wider than the approximately 250 nm full width at half maximum (FWHM) of the narrowest point spread function practically achievable in a confocal microscope (37), imaging methods beyond the diffraction limit are needed to determine subciliary distributions of CEP290 and its binding partners in wild-type and *Cep290*-mutant animals. Given the narrow dimensions of the cilium, we used super-resolution light microscopy — structured illumination microscopy (SIM) and stochastic optical reconstruction microscopy (STORM) (38, 39) — to localize CEP290 precisely within the subcompartments of wild-type and *Cep290*-mutant rod CC, as well as in primary cilia of cultured epithelial cells. We used the same techniques to examine the effects of CEP290 deficiencies on the localization of ciliary components in mouse ciliopathy models. Finally, we correlated our fluorescence localization results with new electron microscopic data revealing ciliary structures at even higher resolution.

## Results

### CEP290 localizes throughout the length of the CC and in close proximity to the microtubule doublets.

**Figure 2:**
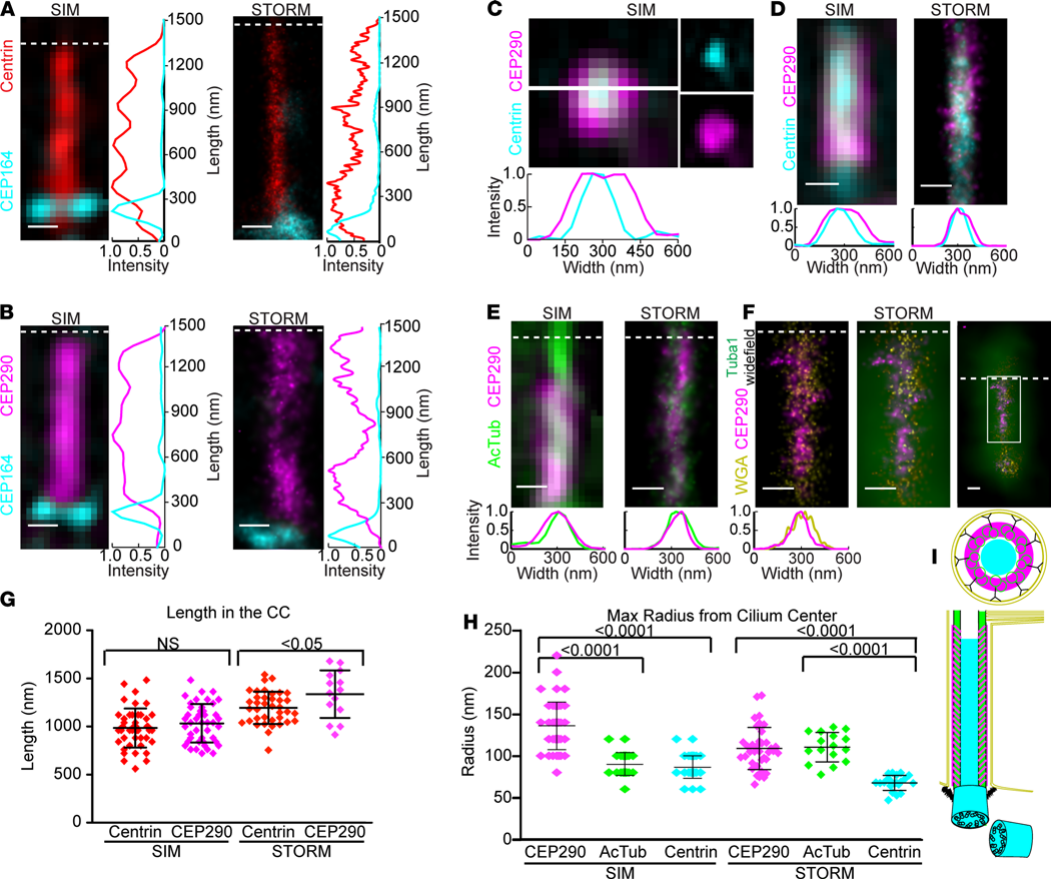
CEP290 localizes throughout the length of the CC and in close proximity to the microtubule doublets. (**A** and **B**) SIM and STORM images of representative cilia from adult retina, immunostained for transition fiber protein CEP164 and axonemal lumen protein Centrin (**A**) or CEP164 and CEP290 (**B**). Row average intensity plots are shown. (**C**) SIM image of a representative cross section through the CC with separated channels to the right. The white line depicts the position of the average intensity line plot. (**D**–**F**) SIM and STORM longitudinal images of representative cilia immunostained for CEP290 and acetylated α-tubulin (AcTub, **E**) or glycocalyx label WGA (**F**). Row average intensity plots are shown. For CEP290/WGA-labeled cilia, high- and low-magnification wide-field Tuba1 antibody staining (green) overlay is shown. (**G**) Dot plot with averages and standard deviations of the lengths of CEP290 and centrin staining in the CC for SIM and STORM. (**H**) Dot plot with averages and standard deviations of the radii of CEP290, AcTub, and centrin in the CC for SIM and STORM. Thirty-three percent of the maximum intensity value of each channel was used as the boundary criterion for the measurement of each cilium. Measurements were compared with Student’s 2-tailed *t* test and 1-way ANOVA and Tukey’s post hoc test, respectively. (**I**) A color-coded schematic of a CC. Scale bar: 200 nm. Dotted line indicates CC/OS border. Measurements were from 3 different animals and SIM images of 75 cilia for CEP290, 30 for AcTub and 45 for centrin, or STORM images of 42 cilia from 6 animals for CEP290, 16 cilia from 3 animals for AcTub and 52 cilia from 3 animals for centrin. AcTub, acetylated α-tubulin; WGA, wheat germ agglutinin; CC, connecting cilium.

To determine the spatial distribution of CEP290 within the CC, mouse retinas and retinal sections from adult animals (6 weeks to 8 months) were colabeled with antibodies for CEP290 and cilia markers with known distributions. To distinguish CC staining from inner segment staining, we used CEP164, a protein whose antibodies label the transition fibers/distal appendages, structures located at the inner segment (IS)/CC interface (40). We used an antibody specific for centrins, a set of calcium-binding proteins known to be centrally localized throughout the length of the CC (27–31, 41), to calibrate the length of the axoneme (Figure 2A). To quantify the extent of CEP290 localization along the length of the CC and to compare it to that of centrin localization, we measured the distances between the proximal and distal boundaries of CEP290 signal (Figure 2B), defined as the positions near the edges where signal was 33% of its maximal value for SIM and STORM, respectively. When measured from the IS/CC interface, marked by CEP164, the average lengths of centrin and CEP290 labeling were similar in SIM: 984 ± 201 nm and 1033 ± 200 nm, respectively. In STORM, the average lengths were 1193 ± 168 nm and 1336 ± 248 nm, respectively (Figure 2G), suggesting that CEP290’s localization may extend to the base of the outer segment, beyond the distal boundary of centrin. The small differences in length between the 2 imaging modalities can be explained by the differential stretching or shrinking of structures in the very different fixation and imaging media used for each method. These values are in agreement with previously reported measurements of CC length (26) and indicate that CEP290 localizes throughout the length of the CC. This finding is in contrast with its localization in other primary cilia, as discussed below, and indicates that the functional role of CEP290 in photoreceptors is performed throughout the entire CC and is not restricted to the base of the transition zone, as it is in the transition zones of other characterized cilia.

To determine the radial distribution of CEP290, we colabeled retinas with antibodies for centrins, which localize to the centermost compartment of the CC; AcTub, which labels the microtubule doublets of the axoneme (42); and wheat germ agglutinin (WGA), a lectin that binds to the glycoproteins of the ciliary membrane (43). Cross sections through the CC imaged with SIM revealed that CEP290 localized outside the region of the lumen (Figure 2C). Longitudinal SIM images (Figure 2, D–F) depict the width of the CEP290 signal as greater than the centrin and AcTub signal widths, while STORM images depict the widths of the CEP290 and AcTub signals as the same but wider than the centrin signal and within the WGA-stained membrane of the CC. Radial measurements of the labeled areas in longitudinal images, measured from the center of the cilium to the position of 33% of maximal signal for SIM and STORM, are shown in Figure 2H. The average maximal radii of centrin and AcTub were 87 ± 14 nm and 91 ± 14 nm, respectively, from SIM images and were 66 ± 9 nm and 109 ± 17 nm, respectively, from STORM reconstructions. These values are in agreement with previously reported radial measurements of the CC (26). The radius of CEP290 staining measured 137 ± 28 nm and 107 ± 25 nm in SIM and STORM, respectively. The small difference may reflect differences in sample preparation as suggested above, possibly leading to differential staining of CEP290 in different subregions of the CC in each method. Interestingly, the distribution of CEP290 signal was, in many cases, not symmetric about the central axis of the CC as would be expected for a protein in solution with a uniform concentration, but rather, was shifted either to the left or to the right of the central axis (Supplemental Figure 1; supplemental material available online with this article; https://doi.org/10.1172/jci.insight.145256DS1). Both the width of CEP290 we observed by STORM and the asymmetry of its staining pattern are consistent with recently reported STORM images collected from cross-sectional views of centrioles in RPE-1 cells (44) but narrower than the approximately 250 nm width observed by stimulated emission depletion (STED) microscopy of longitudinal views in the same cell type (18), which is more consistent with our SIM results. Figure 2I summarizes schematically the longitudinal and radial distribution of CEP290 within the rod CC. These results indicate that CEP290 localizes in close proximity to the microtubule doublets, likely in a regular structural pattern throughout the CC.

### Y-links localize throughout the length of the CC.

**Figure 3:**
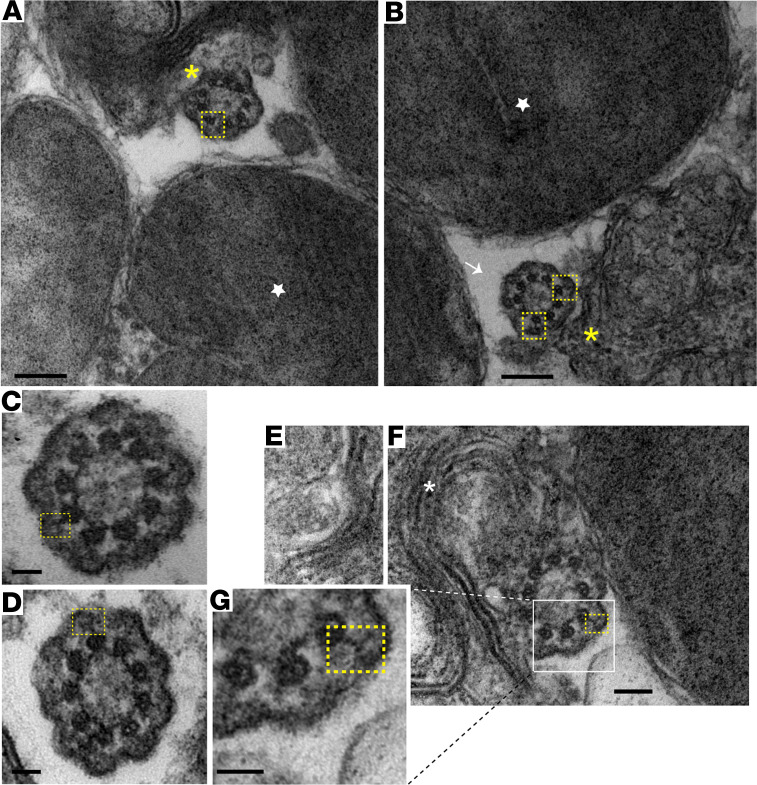
Y-links appear throughout the CC. (**A** and **B**) Transmission electron microscopy (TEM) image depicting the OS/CC interface. CC (white arrow), discs en face (star), and cilium and discs (yellow asterisks) from photoreceptors are shown, with MT doublets connected to Y-links outlined in yellow boxes. Scale bar 200 nm. (**C** and **D**) CC from neighboring photoreceptor cells. Y-links (yellow boxes) are visible. Scale bar: 50 nm. (**E** and **F**) Image of cilium and discs (white asterisk) from another photoreceptor cell. Y-links (yellow box) are visible. Scale bar: 100 nm; inset (**G**) scale bar: 50 nm. CC, connecting cilium; OS, outer segment; MT, microtubule.

Y-links are ill-defined fibrous structures that radiate from each microtubule doublet pair to the ciliary membrane. CEP290 has been proposed to provide structural stability to the transition zone in primary cilia by contributing to or forming the Y-links (45). To investigate whether the Y-links of the photoreceptor CC localize to the same subcellular compartment as CEP290, i.e., throughout the length of the CC and in the region between the microtubules and the membrane, we performed transmission electron microscopy on sections cut as nearly perpendicular as possible to the ciliary axes. We imaged multiple cross-sectional CCs close to or overlapping with the base of the outer segment (Figure 3A). As expected, our images showed Y-links in the proximal CC, identified by the absence of discs and outer segment membrane (Figure 3, B and C). We also found that Y-links were present in the distal CC, a plane identified by the presence of outer segment discs en face, indicating that the sections were not cut obliquely. Present within the same plane were examples in which the fusion of the CC membrane with the outer segment membrane could be seen (Figure 3, D–G). Thus, our data show that Y-links are present throughout the length of the CC, consistent with the longitudinal distribution of CEP290. These findings are also in agreement with previously reported studies using electron microscopic techniques on photoreceptors (46–48), although those did not clearly demonstrate Y-links in the distal CC at the base of the outer segment.

### CEP290 localizes to the base of the ciliary transition zone in nonphotoreceptor primary cilia.

**Figure 4:**
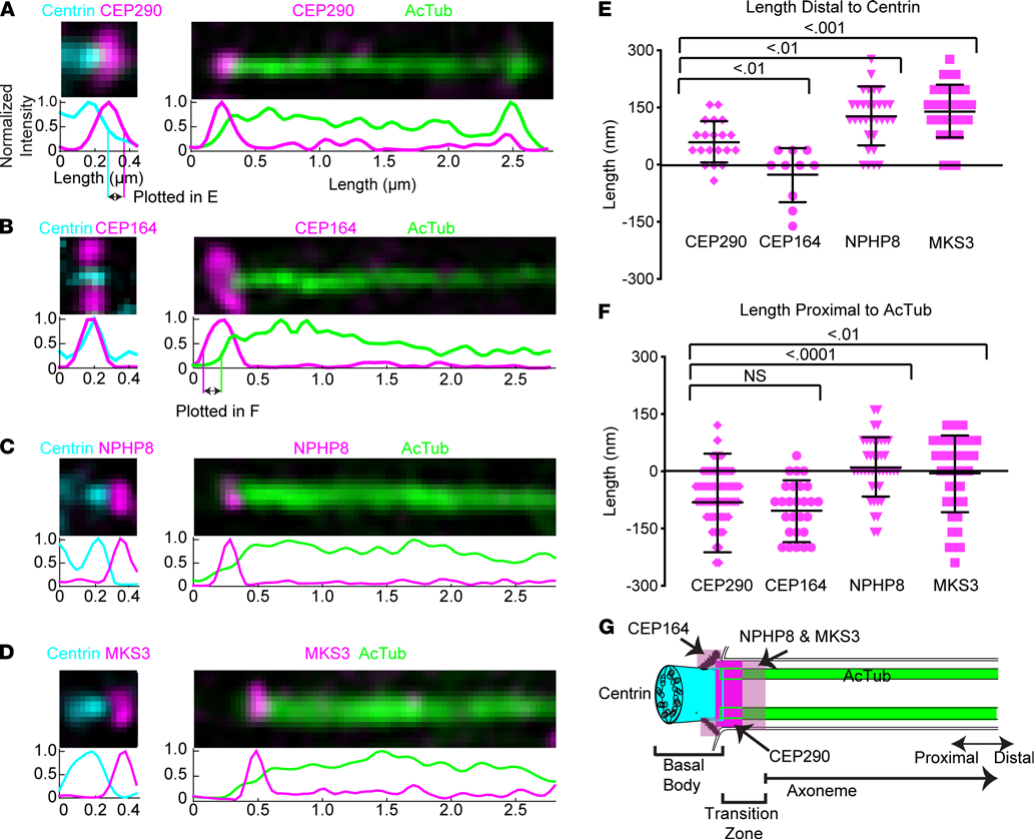
CEP290 localizes to the base of the primary cilium in epithelial cells. (**A**–**D**) SIM images of a representative centriole and cilium for each labeling condition, CEP290, CEP164, NPHP8, and MKS3, respectively, with centrin as a marker for the BB, and AcTub as marker for the axoneme. Row average intensity plots are below each image. (**E** and **F**) Dot plot with average and standard deviation of the length distal to centrin (illustrated in intensity plot of **A**) or the length proximal to AcTub (illustrated in intensity plot of **B**). Thirty-three percent of the maximum intensity value of each channel was used as the boundary criterion for the measurement of each cilium. Measurements were compared with 1-way ANOVA and Dunnett’s post hoc test. (**G**) A color-coded schematic of a primary cilium with results shown. AcTub, acetylated α-tubulin.

**Figure 5:**
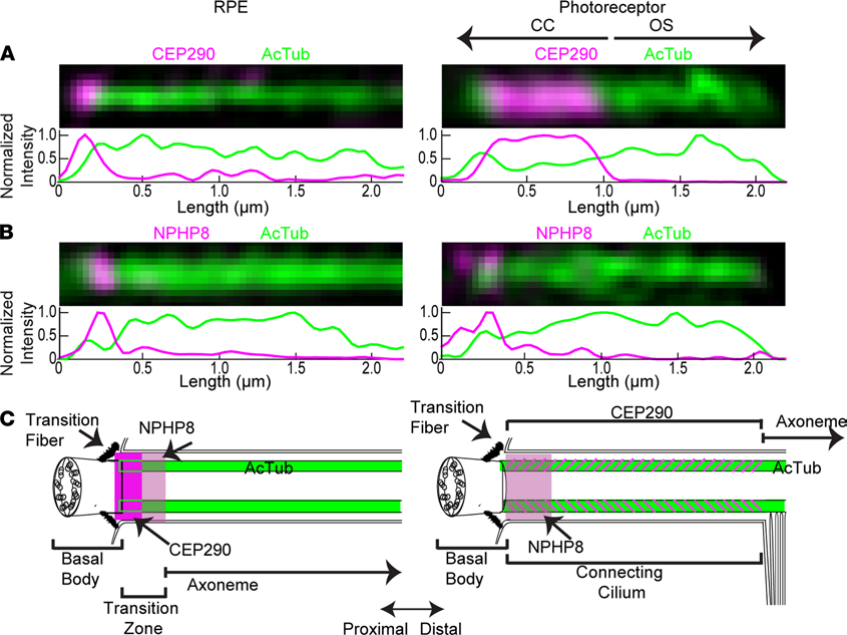
Comparison of ciliary protein localization in RPE and photoreceptor cilia. (**A** and **B**) SIM images depicting CEP290 and NPHP8 localization in RPE and photoreceptor cilia. Row average intensity plots are below each image. (**C**) A color-coded schematic of a primary cilium in RPE and photoreceptor with results shown. See also Supplemental Figure 4. AcTub, acetylated α-tubulin.

To compare CEP290 localization in the CC to that in primary cilia of nonphotoreceptor cells, we examined CEP290 localization in primary cilia of epithelial cells. Human retinal pigment epithelium 1 (hRPE-1) cells are cultured cells that form primary cilia upon serum starvation. Previously, it was reported that CEP290 did not precisely localize with other transition zone proteins in hRPE-1 cells, and that, instead, CEP290 localized between the basal body and the other transition zone proteins (18). We used SIM to image ciliated hRPE-1 cells immunolabeled with centrin antibody to identify the basal body (centrin in hRPE-1 cells is restricted to the basal body and not the lumen of the ciliary axoneme as in rod CC); with AcTub antibody to label the axoneme; with Meckel-Gruber syndrome 3 (MKS3) (18, 49, 50) and nephronophthisis 8 (NPHP8) (18, 51, 52) components of the MKS and NPHP modules, respectively, to identify the distal transition zone; and with CEP164 antibodies to mark the transition fibers/distal appendages, radial structures attached to the distal end of the mother centriole in the BB (44). Consistent with previous reports (18, 33, 44), CEP290 and CEP164 localized below the base of the cilium in hRPE-1 cells (Figure 4, A and B). In both cases, CEP290 and CEP164 labeling appeared more proximal than AcTub labeling (Figure 4, A and B, and Supplemental Figure 4). NPHP8 and MKS3 localized to the cilium and distal basal body (Figure 4, C and D). On average, the distance between the distal edge of CEP290 and the distal edge of centrin was 64 ± 54 nm (*n* = 20) (Figure 4E), while the distance between the proximal edge of CEP290 and the proximal edge of AcTub was –84 ± 129 nm (*n* = 72) (Figure 4F). Thus, the CEP290 signal in hRPE-1 cilia partially overlapped with centrin and AcTub at the base of the cilium. The average extent of CEP164 beyond centrin was –24 ± 71 nm (*n* = 10) (Figure 4E); i.e., the distal edge of CEP164 staining was on average more proximal than the distal edge of centrin staining. The distance between the proximal borders of CEP164 and AcTub was –106 ± 81 nm (*n* = 28) (Figure 4F). CEP164 signal in hRPE-1 cells primarily overlapped with centrin. MKS3 and NPHP8 were 10 ± 78 nm (*n* = 42) and –8 ± 101 nm (*n* = 42) proximal to AcTub labeling, respectively, and 145 ± 70 nm (*n* = 32) and 133 ± 78 nm (*n* = 32) beyond centrin labeling, respectively. The proximal borders of MKS3 and NPHP8 signal in hRPE-1 primarily overlapped with that of AcTub. Figure 4G illustrates schematically CEP290 localization in hRPE-1 primary cilia in relation to centrin, CEP164, MKS3, NPHP8, and AcTub. These results suggest that CEP290 in the primary cilia of epithelial cells is predominantly located at the base of the axoneme, overlapping with but partially distal to CEP164 and centrin, and does not extend throughout the entire transition zone, as demarcated by MKS3 and NPHP8, consistent with previous results obtained by super-resolution fluorescence (18). These results are strikingly different from those obtained in rod cells (Figure 5), supporting the idea of photoreceptor-specific functions for CEP290 in the CC.

These results are also consistent with the hypothesis that CEP290 is associated with the Y-links, because, as shown in a recent electron tomographic study of primary cilia in a different epithelial cell line (IMCD3 cells), structures corresponding to Y-links were observed only within the first 100 nm of the transition zone (21), although various structures connecting the axoneme and membrane were found beyond that point. To further test this idea that CEP290 has distinct localization within the rod CC as compared to its distribution in other primary cilia, we compared the localization of NPHP8, CEP290, and AcTub in primary cilia of hRPE-1 cells and CC (Figure 5 and Supplemental Figure 2) using SIM. In both cell types, CEP290 was distal to the transition fibers (Figure 2, A and B, and Figure 4B); however, in epithelial cell cilia, CEP290 signal was approximately 200 nm in length, whereas in the CC, CEP290 was approximately 1100 nm in length, the full length of the CC. NPHP8 was seen to localize to the proximal end of the cilium in both primary cilia and CC. In primary cilia, NPHP8 localization was distal to CEP290, whereas in the CC, NPHP8 was not distal to CEP290 and did not display localization throughout the entire CC (Figure 5). These results point to the possibilities that CEP290, and other transition zone markers that have been found to localize throughout the length of the CC, such as retinitis pigmentosa GTPase regulator (RPGR) (53), RPGR interacting protein 1 (RPGRIP1), SPATA7, NPHP1, and NPHP4 (36), have a photoreceptor-specific function and that the CC is not simply an expanded transition zone.

### The CC develops in CEP290 mutants prior to retinal degeneration.

We next asked how mutations in *Cep290* affect CEP290 protein localization and CC morphology in rod neurons. LCA is a nonsyndromic retinal disease that results in blindness or severe visual impairment in humans within the first year of life. One intronic *Cep290* mutation that leads to insertion of a cryptic stop codon and protein truncation at position 998 accounts for roughly 20% of LCA cases (6). *Cep290^rd16/rd16^* (hereafter referred to as *Rd16*) mice also display signs of a nonsyndromic retinal disease and, thus, are commonly used as a model for LCA (54, 55). The *Rd16* allele contains an in-frame 300–amino acid deletion that overlaps with the putative microtubule binding domain of CEP290 (56). *Rd16* mice undergo rapid photoreceptor degeneration and develop abnormal, rudimentary outer segments prior to degeneration. There are 2 other CEP290 mutant mice we evaluated in this manuscript: a *Cep290^tm1.1Jgg^* mutant and a knockout (KO) of CEP290. The *Cep290^tm1.1Jgg/tm1.1Jgg^* mouse is a model for Joubert syndrome (57) with rapid photoreceptor degeneration and vermal hypoplasia (57). Joubert syndrome is a syndromic ciliopathy characterized by nephronophthisis, cerebellar vermis aplasia, and retinal degeneration (58). These *Cep290^tm1.1Jgg/tm1.1Jgg^* animals will be referred to throughout the rest of the paper as a near null (NN), because an alternatively spliced variant of the mutant allele may result in low residual levels of a truncated CEP290 (59). The *Cep290*-knockout (*Cep290^tm1Asw/tm1Asw^*, hereinafter referred to as KO) mouse is also a model for Joubert syndrome and was generated by inserting a β-gal-neo^R^ cassette in place of exons 1–4 in the *Cep290* allele. The KO mice have hydrocephalus, rapid retinal degeneration, mild renal disease, and premature death (60).

To determine whether mutations in or loss of *Cep290* affects localization of CEP290 or other CC proteins, we assessed age-matched mutants and wild-type (WT) animals using super-resolution microscopy. Since photoreceptor discs begin to form at postnatal day ~7 (P7) and the *Rd16* mutant animals undergo photoreceptor cell death as early as P14 (55, 60) (Supplemental Figure 3), we used P10 animals to assess CC protein localization prior to photoreceptor degeneration; we found that this age provided the best balance between formation of cilia and cell death (Supplemental Figure 3).

**Figure 6:**
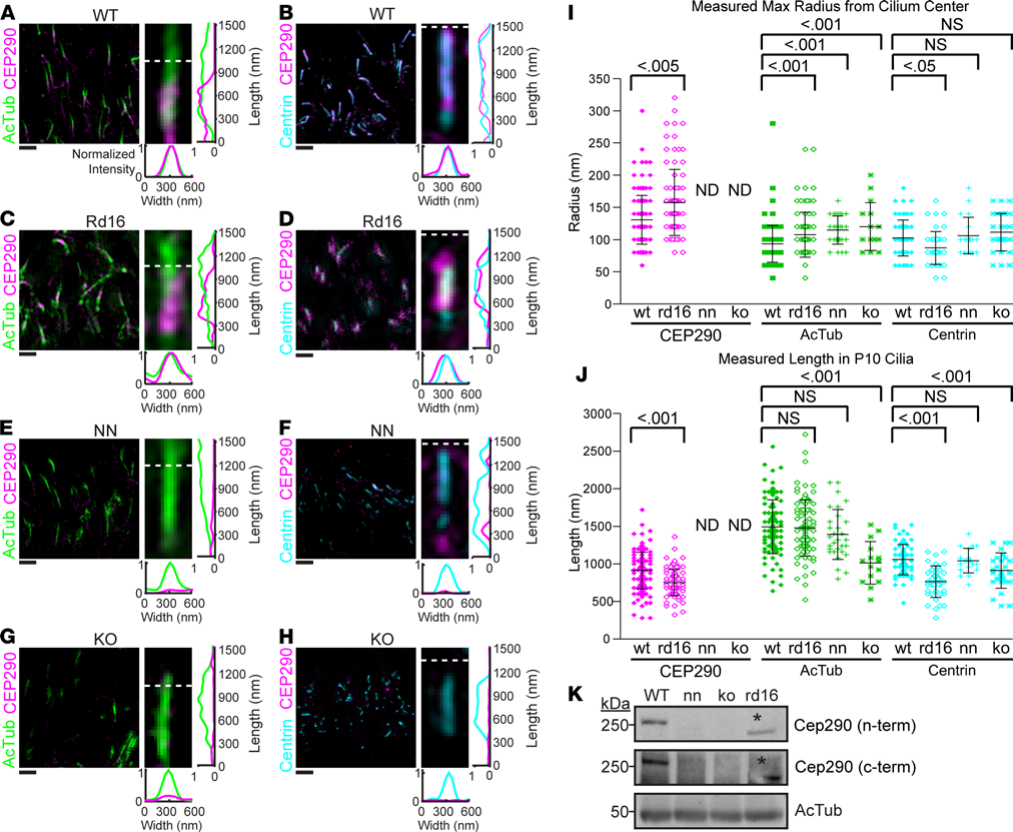
CC develops in *Cep290* mutant prior to degeneration. (**A**–**H**) SIM images (high magnification, right; low magnification, left) of cilia from P10 WT, rd16, NN, and KO animals labeled with CEP290, AcTub, and centrin. Scale bar on left: 1 μm. SIM (right) images of representative cilia. Row average intensity and column average intensity plots are shown. AcTub and centrin gains were adjusted to subsaturation for image presentation. CEP290 intensity levels in Cep290 mutants were normalized to WT levels. (**I** and **J**) Dot plots with averages and standard deviations of the maximum radii and lengths of CEP290, AcTub, and centrin in the cilium. Cilia were imaged from 3 nonlittermate mice for each genotype. Thirty-three percent of the maximum intensity value of each channel was used as the boundary criterion for the measurement of each cilium. Measurements were compared with 1-way ANOVA and Dunnett’s post hoc test. (**K**) Western blots demonstrating detection of CEP290 protein products. Expected sizes for WT CEP290, CEP290 (rd16), and AcTub are 290 kDa, 270 kDa, and 50 kDa, respectively. The N-terminal blot lanes were run simultaneously on the same samples as the C-terminal blot. Asterisk indicates band shift in the rd16 protein product. Dotted line indicates CC/OS border. Full blot for **K** is available in the supplemental materials. AcTub, acetylated α-tubulin; CC, connecting cilium; WT, wild-type; rd16, *Cep290^rd16^*; nn, near null-*Cep290^tm1.1Jgg^*; ko, *Cep290*-knockout; ND, not detectable.

Surprisingly, at this early age, the CC markers we tested were only mildly mislocalized in the *Rd16*, NN, and KO mutant rods compared with WT. Centrin was localized throughout the CC lumen in WT and mutant cilia (Figure 6, B, D, F, and H), although the length of centrin staining was generally shorter in mutant CCs. AcTub labeling of microtubule doublets in the CC and axoneme in WT rod cilia (Figure 1, B and C, and Figure 6A) was similar in the CC and the rudimentary outer segments of the mutant retinas (Figure 6, C, E, and G).

On the basis of C-terminal CEP290 antibody labeling, CEP290 protein was localized longitudinally throughout the CC and radially between the axoneme and the ciliary membrane of WT and mutant *Rd16* rods (Figure 6, A–D), indicating that the missing CEP290 domain was not essential for CEP290 localization. We observed little or no labeling with the C-terminal CEP290 antibody in NN or KO retinas at P10 (Figure 6, E–H); this finding was supported with Western blotting (Figure 6K; see complete unedited blots in the supplemental material).

To quantify the difference in distributions of centrin, AcTub, and CEP290, we measured the length and radius of antibody labeling in mutant and age-matched WT controls. Although P10 animals have developed CC, ciliogenesis is not yet complete at this age, and the ciliary dimensions are not necessarily identical to those of adult mice. For radial measurements, there were small but significant differences between WT and *Cep290* mutant rod cilia (Figure 6I). Centrin measurements were 102 ± 28 nm (*n* = 70), 87 ± 26 nm (*n* = 45), 106 ± 28 nm (*n* = 20), and 111 ± 29 nm (*n* = 30) for WT, rd16, NN, and KO, respectively (Figure 6I). There was a small but significant difference between WT and rd16 (*P* < 0.05, 1-way ANOVA with Dunnett’s post hoc analysis). However, we found no significant difference between WT and either NN or KO (*P* > 0.05). AcTub measurements were 93 ± 28 nm (*n* = 107), 107 ± 35 nm (*n* = 90), 115 ± 22 nm (*n* = 30), and 120 ± 37 nm (*n* = 15) for WT, rd16, NN, and KO, respectively (Figure 6I). AcTub was significantly wider in all *Cep290* mutants compared with WT (*P* < 0.001). Given that TEM results suggested a slight contraction of the axonemal radius in CEP290 mutants (see below), the wider appearance of AcTub staining may reflect a greater tendency of the axoneme to flattening in the image plane of the CEP290 mutant retinas.

CEP290 measurements were 131 ± 38 nm (*n* = 135) and 158 ± 51 nm (*n* = 89) for WT and rd16, respectively, and CEP290 immunolabeling in rd16 CC was significantly wider than in WT (*P* < 0.005). CEP290 immunolabeling in NN and KO was not detectable.

Similarly, in longitudinal measurements, there were small but significant differences between WT and *Cep290* mutant rod cilia (Figure 6J). Rd16 and KO cilia were significantly shorter than WT when measured with centrin labeling (*P* < 0.001). However, no difference was found for WT versus NN (*P* > 0.05). AcTub labeling appeared slightly shorter in the mutants; however, only the KO cilia achieved statistical significance compared with WT (*P* < 0.001). CEP290 measurements were 913 ± 244 nm (*n* = 135) and 750 ± 175 nm (*n* = 89) for WT and rd16, respectively, which were significantly shorter than WT (*P* < 0.001) (Figure 6J). Overall, *Cep290*-mutant cilia tended to be wider and shorter compared with age-matched WT cilia. The width differences were consistent with observations by electron microscopy (see below).

Because the N-terminus of CEP290 is proposed to interact with the ciliary membrane and reported to have a slightly wider radial extent than the C-terminus (61, 62), we also used an N-terminal CEP290 antibody to immunolocalize CEP290 in WT animals. The radial width of our N-terminal CEP290 staining was not much wider than the C-terminal CEP290 staining, and the difference was not statistically significant (Supplemental Figure 2). Nonetheless, the resolution of these experiments does not allow us to rule out the possibility of CEP290-membrane interactions in vivo.

Since the rd16 deletion in CEP290 protein affects the putative microtubule binding domain of CEP290 (56), we asked whether the localization of CEP290 in relation to AcTub is affected in the *Rd16* cilia. The average distances between these antigens in the 2 genotypes differed from one another by less than the pixel size of 40 nm (*n* = 50 for WT, and *n* = 48 for rd16 cilia) and therefore are not reliably resolvable by SIM.

### Functional CEP290 is not required for CC formation.

We next tested the localization of the proposed CEP290 binding partner NPHP5 in mice of different genotypes. *NPHP5/IQCB1* is a causal gene of LCA and Senior-Löken syndrome (SLS) (63–65), and LCA and SLS patients with NPHP5 mutations phenocopy *CEP290*-LCA and *CEP290*-SLS cases (55). The C-terminal region of NPHP5 binds to the N-terminal region of CEP290 (66), forming a complex that, through unknown mechanisms, regulates protein trafficking in primary cilia of IMCD3 and hRPE-1 cells (66, 67).

**Figure 7:**
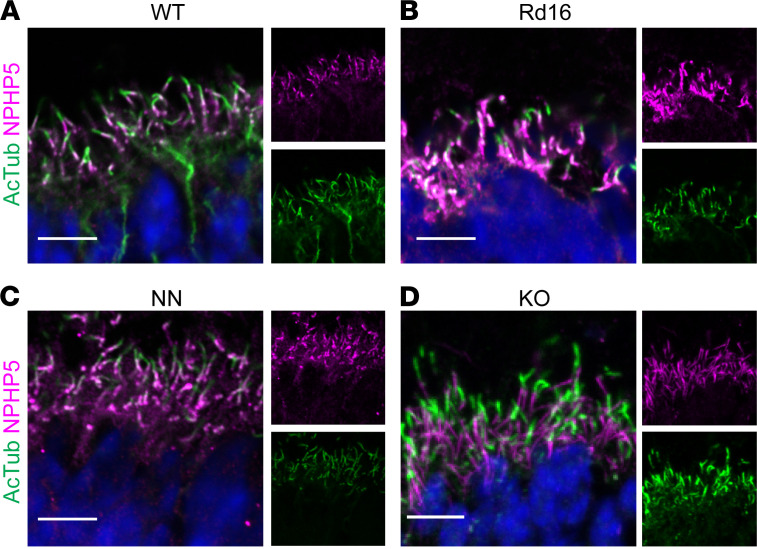
NPHP5 localization in WT and in *Cep290* mutants. NPHP5 antibody signal localizes to the base of the CC and the region on the rootlet and BB in WT and in Cep290 mutant animals before photoreceptor degeneration. Confocal images of P10 WT (**A**), rd16 (**B**), NN (**C**), and KO (**D**) cilia with separated channels at low magnification to the right. WT image acquisition and processing settings were applied to WT, rd16, NN, and KO data. Scale bar: 10 μm. AcTub, acetylated α-tubulin; CC, connecting cilium; WT, wild-type; rd16, *Cep290^rd16^*; nn, near null-*Cep290^tm1.1Jgg^*; ko, *Cep290*-knockout.

We observed NPHP5 localization in the region of the rootlet, basal body, and base of the CC at P10 in all genotypes (Figure 7, A–D). Previous work using a different NPHP5 antibody (64) and different fixation conditions suggested somewhat different patterns of NPHP5 staining, with signal throughout the CC and in the OS (64) or in the proximal OS (68). It is also reported that in NPHP5^–/–^ mice, CEP290 is extensively mislocalized in the ONL and synaptic terminals, a much more dramatic effect than the CEP290 KO has on NPHP5 localization. Although near or complete loss of CEP290 in our mutant mice results in rapid retinal degeneration and severe phenotypes throughout multiple tissues, nascent photoreceptors in the mutant retina are still able to form cilia with at least a subset of normally localized CC proteins.

**Figure 8:**
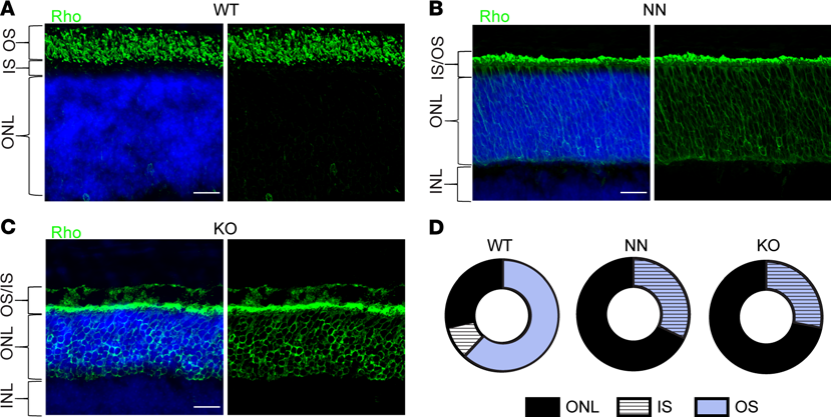
Rhodopsin localization in P10 *Cep290* mutant cilia. (**A**–**C**) Confocal images of retina from P10 WT, NN, and KO stained for rhodopsin (green) and DAPI (nuclear stain, blue). Acquisition settings and image processing were identical for all samples. Scale bar: 20 μm. (**D**) Integrated intensities for each layer normalized by total rhodopsin signal for the images above. OS, outer segment; IS, inner segment; ONL, outer nuclear layer; INL, inner nuclear layer; WT, wild-type; rd16, *Cep290^rd16^*; nn, near null-*Cep290^tm1.1Jgg^*; ko, *Cep290*-knockout.

We also tested the localization of rhodopsin, the visual pigment of the rod photoreceptors, in our mutant mice. Rhodopsin is the most abundant protein in these cells, accounting for up to 90% of total protein in disc membranes of the outer segment (69, 70). We used immunofluorescence with rhodopsin antibodies in retinal sections from P10 NN and KO mice to determine if normal rhodopsin outer segment localization was impaired. While we observed rhodopsin colocalized with cilia at the distal end of nascent photoreceptors in the NN and KO retinas, rhodopsin was also mislocalized in plasma membrane of the IS and the ONL in both mutants, as compared with WT retinas (Figure 8). One possible cause could be rhodopsin protein overloading the underdeveloped outer segment in CEP290-deficient rods. Rhodopsin mislocalization, along with the small changes in radial and longitudinal distribution of CC proteins, are the earliest structural or functional defects yet observed in CEP290-mutant photoreceptors and may be the basis for the later defects in outer segment development and cell survival.

### Y-links are present in Cep290-mutant CC.

**Figure 9:**
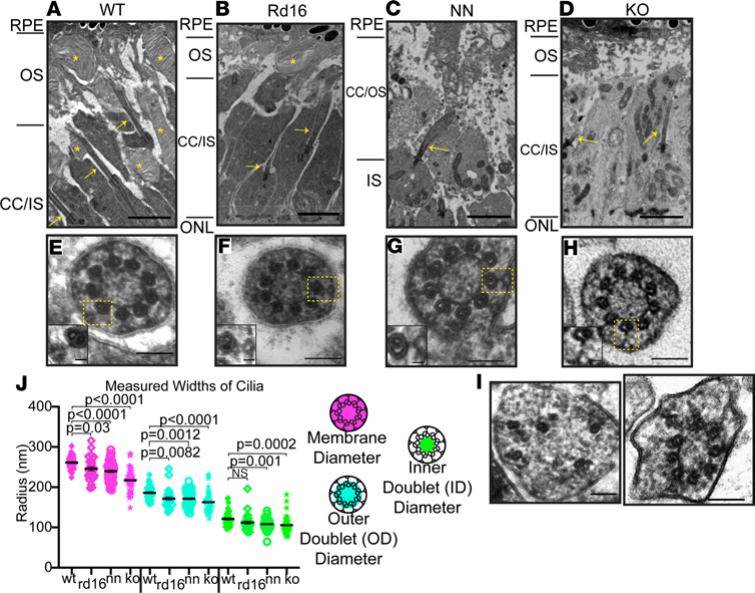
Y-links are present in P10 *Cep290* mutant CC. TEM longitudinal images of P10 photoreceptor cilia from (**A**) WT, (**B**) *Rd16*, (**C**) NN, and (**D**) KO animals depicting the (**A**) properly developed CC and OS, (**B**) rudimentary OS, and (**C** and **D**) rudimentary CC. CC (yellow arrow) and OS discs (yellow asterisk) are highlighted. Scale bar: 2 μm. (**E**–**H**) Cross section images through the CC of (**E**) WT, (**F**) *Rd16*, (**G**) NN, and (**H**) KO animals. Y-links (yellow box) are visible in WT and *Cep290* mutants. Scale bar: 100 nm. Insets highlight Y-links within the box. Scale bar: 20 nm. Lower magnification views of the same regions of these retinas are shown in Supplemental Figure 6. (**I**) Cross sections through distal ends of the CC showing abnormal structures in KO retinas. See also Supplemental Figure 5B. (**J**) Dot plot with averages and SEMs of measurements of annotated areas within CC sections from WT (43 cilia from 2 animals), *Rd16* (29 cilia from 3 animals), NN (58 cilia from 2 animals), and KO (44 cilia from 2 animals). Measurements were compared using 1-way ANOVA with Dunnett’s test.

Because we could detect CC by immunofluorescence in developing rods expressing a mutant form of CEP290 at roughly normal levels (*Rd16*), an aberrant form at substantially reduced levels (NN), or no detectable CEP290 (KO), we next asked whether these *Cep290*-mutant CC had Y-links. We used TEM to capture longitudinal and cross sections through rod CC from P10 WT, *Rd16*, NN, and KO retinas. In longitudinal TEM images, we found that *Cep290*-mutant rods formed mostly normal cilia compared to WT cilia (Figure 9, A–D), though at reduced numbers in the NN and KO sections. *Rd16* rods formed CC and rudimentary outer segments (Figure 9B), while NN rods formed rudimentary CC and a small number of nascent outer segment disc-like structures (Figure 9C). We observed almost no disc-like structures in KO TEM sections (Figure 9D). Lower magnification TEM images of longitudinal sections through the P10 *Cep290*-mutant retinas displayed overall normal retinal organization outside the photoreceptor ciliary region in all genotypes (Supplemental Figure 6).

In cross sections of the CC, examples from all genotypes had densities that radiated from the microtubule doublets and widened at the ciliary membrane in a manner similar to the Y-links (Figure 9, E–H). Although the structures of the CC in the *Cep290* mutants appeared grossly normal, we carefully measured the CC dimensions in our TEM cross sections (as depicted schematically below each graph, Figure 9I). We found small contractions of both the axoneme and the ciliary membrane in the mutant CC (Figure 9I). The diameters for the axoneme were 161.3 ± 3.4 nm in KO, 171.6 ± 2.4 nm in NN, and 171.5 ± 4.3 nm in *Rd16* compared with 186 ± 2.5 nm in WT. The diameters for the ciliary membrane were 217.3 ± 4.4 nm in KO, 239.7 ± 3.1 nm in NN, and 245.8 ± 5.2 nm in *Rd16* compared with 261.1 ± 2.4 nm in WT. Note that we measured diameter in our TEM data by taking 2 perpendicular radius measurements of cross sections and used the shortest one in our analysis; this procedure is in contrast to our fluorescence measurements, in which the widths of longitudinal sections are measured; these tend to yield results wider than the actual diameter as the cilia tend to flatten somewhat in the imaging plane. Interestingly, all 3 mutant retinas displayed Y-link structures in the CC (Figure 9, E–H). Cross-sectional views were difficult to find in the KO. Not all had obvious Y-links, but many did. These results suggest that a fully functional CEP290 is not required to form the Y-links and is not an essential structural component of the Y-links. However, CEP290 may be required for the Y-links to form uniformly throughout the length of the CC, and CEP290 may function indirectly for proper Y-link assembly or stabilization. Additionally, CEP290 in conjunction with the Y-links may be integral to the structural integrity of the CC, since the distal CC in the KO (a) did not usually contain Y-links, (b) did not hold the canonical shape, and (c) did not contain some other CC structures, e.g., the central ring (Supplemental Figure 5).

## Discussion

The results presented here address the localization of CEP290 and its influence on the localization of other proteins in rod cilia and have important implications for its function and for the mechanisms of disease caused by CEP290 deficiencies. The observation that CEP290 is distributed throughout the length of the rod CC, yet is confined to a narrow region of approximately 200 nm at the base of epithelial cell cilia, suggests its function may also be different in these distinct cell types. Its close association with the axoneme is consistent with another report (62) and with proposals for its contributing to structural connections between microtubules and the membranes, as is the frequently asymmetric distribution of CEP290 with respect to the central axis of the cilium.

Based to some extent on lower resolution localization methods, there have been suggestions that what distinguishes the CC from the proximal portions of other cilia is primarily length — that CC could be considered simply a long transition zone. However, this interpretation would be an oversimplification of the multiple unique features of the CC. Whereas CEP290 is located throughout the length of the CC, it is not located throughout the transition zone of other cilia, either in primary cilia, such as the hRPE-1 cells studied here and by others (18, 44), or in motile cilia, such as those in *Chlamydomonas reinhardtii* (71), but is rather confined to a narrow subcompartment of the transition zone (TZ) and could be considered a component of the distal end of the mother centriole in those cells. Interestingly, in motile cilia of *Paramecium* (72), CEP290 was reported to reside in a distal subcompartment of the transition zone, in close proximity to NPHP8 and transmembrane protein 107 (TMEM107), but more distal than other TZ components, NPHP4 and TMEM216, and to facilitate ciliary shedding induced by Ca^2+^ and ethanol. Centrins are not observed in transition zones of hRPE-1 cell primary cilia (Figure 4) but are found throughout the length of the CC. Some essential CC-resident proteins, such as RPGR^orf15^ (73) and RPGRIP1 (74), localize to centrioles in other cell types (75), and others, such as SPATA7 (36), are missing or nonessential in TZ of other cilia. It was recently reported (76) that MKS3/TMEM67, which is considered a marker of the TZ in primary cilia (18, 49, 50), is found at the base of and throughout the plasma membrane of rod outer segments, suggesting photoreceptor-specific location and function, which bears further investigation. We show here that the well-characterized TZ marker, NPHP8, locates to a narrow region at the base of the CC, as in other cilia, and not throughout the entire CC length (Figure 5). Therefore, there may be other unique features and protein complexes within the CC required for photoreceptor function that have yet to be elucidated.

In this study, we used 2 different super-resolution imaging techniques — SIM and STORM — to localize CEP290 and other CC proteins within the rod photoreceptor CC of adult mice and young *Cep290*-mutant mice. Each of these techniques, along with other super-resolution methods, such as STED (18, 77) microscopy, cryo-electron tomography (26), or immunoelectron microscopy, has its advantages, but all are also susceptible to distortions and artifacts, necessitating the use of multiple imaging modalities to reach reliable conclusions. Although we were able to successfully image and measure protein distribution in the CC, SIM and STORM produced slightly different numbers for dimensions of labeled regions in the CC. While both techniques provide better resolution than conventional light microscopy, with STORM providing the better resolution of the two, the discrepancies between the imaging modes can be attributed in part to different sample preparations and staining protocols; these results highlight a need for continued improvement in microscopy resolution. Advances in correlated light and electron microscopy, immunogold electron microscopy and expansion microscopy (78–81) show promise, as techniques are improved for allowing the preservation and visualization of the ultrastructure while maintaining the antigenicity necessary for immunolabeling.

Despite the major disruptions in CC structure and rapid retinal degeneration caused by the CEP290 mutations, their effects on the initial stages of cilium formation and localization of other ciliary proteins appear to be less severe. These results are surprising, given suggestions that CEP290 is essential for ciliogenesis and proposals that it plays a major role in localizing NPHP5 and other TZ proteins (33, 67, 82, 83). They are in stark contrast to those obtained for another LCA-associated protein, SPATA7, deficiencies in which cause drastic redistribution of CC proteins, including CEP290 (36). The idea that CEP290 is essential for ciliogenesis in the retina may have arisen from failure to look at early time points in the development of the CC as we have done in this study.

Our results are consistent with another proposed role for CEP290, participation in the Y-link structures connecting the axoneme to the ciliary membrane (61). We found that both these Y-links and CEP290 were distributed along the length of the CC. A recent electron tomographic study of primary cilia in epithelial cells (21) demonstrated that their Y-links radiated outward from the microtubule doublets in a narrow proximal portion of the axoneme, well within the 100 nm of CEP290 labeling we observed at the base of the primary cilium in cultured epithelial cells.

However, our results are not consistent with the idea that it is a major structural component of the Y-links, as they do form in each mutant, albeit it in reduced numbers. This irregular formation of Y-links could contribute to the altered CC dimensions we recorded in *Cep290*-mutant rods (Figure 6, I and J, and Figure 8I). A previous study of a CEP290 mutation in *Chlamydomonas reinhardtii* (45) reported a reduction in the number of Y-links but not obvious abnormalities in structures of those that were present. There were also alterations in the microtubule-to-membrane distances in that mutant, but in that case the distance was increased. All these results support the idea that CEP290 is a component of, or major interactor with, the Y-links and plays an important role in stabilizing the cilium, but CEP290 is not the major Y-shaped structural component bridging the axoneme and ciliary membrane. While we are now able to place additional constraints on CEP290 functions, current evidence suggests CEP290 likely has several, including both those common to cilia generally and those specific for the CC.

## Methods

### Animals.

WT mice used for this study were C57BL/6 aged P10 to 8 months. *Rd16* and *Cep290^tm1.1Jgg^* mice backcrossed to C57BL/6 were acquired from The Jackson Laboratory (*Rd16* stock: 012283; *Cep290^tm1.1Jgg^* stock: 013702). Cep290-KO mice (*Cep290^tm1A/tm1Asw^*) were acquired from Anand Swaroop at the National Eye Institute, NIH, Bethesda, Maryland, USA. Details of how these mice were made and genotyping protocols are published (60). Briefly, exons 1–4 of the *Cep290* gene were replaced with a β-gal-neo^R^ cassette by homologous recombination in embryonic stem cells, thereby creating a null allele. *Cep290*-mutant animals age P10 were used for this study. For immunostaining and immunoblotting, samples from at least 3 replicate mice were used for each genotype. Heterozygous *Cep290^tm1.1Jgg^* crosses were bred to produce *Cep290^tm1.1Jgg^* mice. Genotyping is described under Supplemental Methods. All procedures were approved by the Baylor College of Medicine Institutional Animal Care and Use Committee and adhered to the Association of Research in Vision and Ophthalmology (ARVO) guidelines for the humane treatment and ethical use of animals for vision research.

### Cell culture.

Human hTERT-RPE-1 (hRPE-1) cells were grown in 50/50 Dulbecco’s modified Eagle medium (DMEM)/F12 supplemented with 10% FBS and 10 μg Hygromycin B at 37°C in a humidified 5% CO_2_ atmosphere. To induce ciliation, cells were grown to 70% confluence, split 1:1 in normal growth media on 1.5 coverslips, and allowed to settle for 12 hours prior to changing to starvation media (DMEM/F12 with 0.5% FBS). Cells remained in starvation media for 36 hours.

Cells were fixed with 2% paraformaldehyde (PFA) diluted in 1× PBS at room temperature for 10 minutes, blocked in 1× PBS with 1% BSA and 0.1% Triton X-100 at room temperature for 30 minutes, incubated with primary antibody in blocking buffer for 45 minutes at room temperature, and incubated with secondary antibody in blocking buffer for 30 minutes. Coverslips were mounted with Prolong Glass (Thermo Fisher Scientific).

### Antibodies.

Details of antibodies used for immunofluorescence staining or immunoblotting are provided in Supplemental Methods.

### Sample preparation for confocal, deconvolution, and SIM immunofluorescence microscopy.

Eyecups from mice aged 4–8 months (Figures 2–5) or P10 (Figures 6, 7, and 9) were fixed for 5 minutes in 1% PFA (Electron Microscopy Science) diluted in 1× PBS, cryopreserved in 30% sucrose overnight at 4°C, embedded in optimal cutting temperature media, and flash-frozen by liquid nitrogen. They were further processed for staining and imaging as described in Supplemental Methods.

### Deconvolution microscopy and SIM imaging and image analysis.

For deconvolved wide-field microscopy and SIM, sections were imaged on a DeltaVision OMX Blaze v4 (GE Healthcare, now Cytiva) equipped with 405 nm, 488 nm, 568 nm, and 647 nm lasers and a BGR filter drawer; a PLANPON6 60×/NA 1.42 (Olympus) using oil with a refractive index of 1.520; and front illuminated Edge sCMOS (PCO). For SIM, a total of 15 images were acquired per section per channel at a *Z*-spacing of 125 nm. Deconvolved images were acquired in conventional mode, while SIM images were acquired in SI mode. Reconstructions were performed in Softworx 7 software.

Images were deconvolved and aligned, and SIM images were reconstructed using SI reconstruction and OMX alignment. Default deconvolve and reconstruction settings were used. After analysis, reconstructions were processed in Fiji/ImageJ (NIH), and the Straighten tool was applied to straighten curved or bent cilia to acquire accurate profiles. Regions of interest (ROIs) of digitally straightened deconvolved and STORM reconstructions were measured using row average profiling, which plots the average intensity across the width of the ROI for each row of pixels along the length of the ROI. Pixels were converted to nanometers for accurate scaling. From these row average profiles, the edges of antibody labeling in SIM were set as 33% of the maximum intensity value for connecting cilia and FWHM for hRPE-1 primary cilia, and the radius was measured from the position of maximum intensity value for either AcTub or centrin to the position of 33% maximum labeling for the protein of interest. All measurements were made in a 1.1 μm longitudinal region just above the basal body that corresponds to the length of the ultrastructural CC and provided as mean ± standard deviation. For radius measurements using SIM images, signals that extended beyond either the AcTub or centrin labeling were measured. All measurements were rounded to the nearest nanometer. Measurements were not collected for deconvolved images, due to their poor resolution in the focal plane.

### TEM.

Adult mice were euthanized by CO_2_ asphyxiation followed by decapitation. Eye globes were enucleated and fixed in 0.1 M sodium cacodylate buffer (pH 7.2) containing 2.5% glutaraldehyde at room temperature for 10 minutes. Cornea and lens were removed from the globe, and the fixation of the remaining eyecup continued for 2 hours. After rinsing in buffer, the eyecups were postfixed and heavy metal–contrasted with potassium ferrocyanide, osmium tetroxide, thiocarbohydrazide, uranyl acetate, and lead aspartate. Next, the eyecups were dehydrated in acetone and embedded in EMbed 812 resin (Electron Microscopy Sciences). Serial block face imaging of the resin blocks was performed on a scanning electron microscope (SEM; Mira 3, Tescan) equipped with an in-chamber ultramicrotome (3View, Gatan). Serial images of the sectioned block face (200 nm between sections) were observed on a digital monitor until the tissue plane was reached containing the outer segment–CC interface of the photoreceptors. The sectioned block was then removed from the SEM and placed in a conventional ultramicrotome for routine sectioning (80–100 nm thick) and collection on copper grids (200 mesh) for imaging on the transmission electron microscope (Tecnai 12, FEI). Optimal cross-sectional images of the cilia were obtained using a goniometer specimen chamber capable of ±60° tilting in conjunction with a motorized rotating (360°) specimen holder.

For P10 studies, mice were euthanized by CO_2_ asphyxiation followed by decapitation at P10. Eyes were enucleated, the cornea and lens were removed, and eye globes were placed in fixative (2% PFA, 2% glutaraldehyde, 3.4 mM CaCl_2_ in 0.2 M HEPES, pH 7.4) for 24 hours, rocking at room temperature. The eyecups were then washed in 1× PBS for 5 minutes, placed in 4% agarose, and left to polymerize for 30 minutes at 4°C. Then 150 μm sections were cut on a vibratome and subsequently stained with 1% Tannic Acid with 0.5% Saponin in 0.1 M HEPES, pH 7.4, for 1 hour, with rocking at room temperature. After rinsing in MilliQ water (MilliporeSigma), the sections were stained with 1% uranyl acetate in 0.2 M Maleate Buffer, pH 6.0, for 1 hour, with rocking at room temperature. The sections were rinsed in MilliQ water and dehydrated in a series of ethanol washes (50%, 70%, 90%, 100%, 100%) for 15 minutes each, followed by infiltration with Ultra Bed Epoxy Resin (Electron Microscopy Sciences). The sections were embedded in resin between 2 ACLAR sheets sandwiched between glass slides in a 60°C oven for 48 hours. At 24 hours, the top slide and ACLAR sheet were removed, and resin blocks in BEEM capsules (Electron Microscopy Sciences) were stamped onto each section to allow for polymerization the following 24 hours. Ultrathin silver sections were placed on copper grids and poststained in 1.2% uranyl acetate in MilliQ water for 6 minutes, followed by staining in Sato’s lead (a solution of 1% lead acetate, 1% lead nitrate, and 1% lead citrate; all from Electron Microscopy Sciences) for 2 minutes. Sections were imaged on a Hitachi H7500 or a JEOL JEM-1400 electron microscope. Measurements from radial cross sections of photoreceptor CC were performed on ImageJ. Since the cross sections were not perfectly circular, 2 intersecting measurements (along the shortest and longest axes) were taken, and the shortest measurement was used in the analysis for Figure 9J.

### STORM immunohistochemistry and resin embedding.

Retinas from 6- to 8-week-old WT mice were immunolabeled for STORM using a protocol we developed previously (22). Details are provided in Supplemental Methods.

### STORM image acquisition.

Immediately prior to imaging, 10% sodium hydroxide (*w/v*) was mixed with pure 200-proof ethanol for 45 minutes to prepare a mild sodium ethoxide solution. Glass-bottom dishes with ultrathin retina sections were immersed for 30–45 minutes for chemical etching of the resin. Etched sections were then washed and dried on a 50°C heat block. The following STORM imaging buffer was prepared: 45 mM Tris (pH 8.0), 9 mM NaCl, and oxygen scavenging system: 0.7 mg/mL glucose oxidase (Amresco) + 42.5 μg/mL catalase (MilliporeSigma), 10% (*w/v*) glucose + 100 mM MEA (i.e., l-cysteamine, Chem-Impex) + 10% VECTASHIELD (Vector Laboratories). Imaging buffer was added onto the dried, etched sections and sealed with a second number 1.5 coverslip for imaging.

Imaging was performed on the Nikon N-STORM system, which features a CFI Apo TIRF 100× oil objective (NA1.49) on an inverted Nikon Ti Eclipse microscope. STORM image acquisition was controlled by NIS-Elements Ar software.

To begin a STORM acquisition, both the 561 nm and 647 nm laser lines were increased to maximum power to photobleach the fluorescence and initiate photoswitching. Imaging frames were collected at approximately 56 frames per second. A total of 50,000 frames were collected for each imaging experiment.

### STORM image analysis.

Two-dimensional (2D) STORM analysis of STORM acquisition frames was performed using NIS-Elements Ar Analysis software. Analysis identification settings were used for detection of the individual point spread function (PSF) of photoswitching events in frames from both channels to be accepted and reconstructed as 2D Gaussian data points. These settings were as follows: minimum PSF height: 400, maximum PSF height: 65,636, minimum PSF width: 200 nm, maximum PSF width: 700 nm, initial fit width: 350 nm, maximum axial ratio: 2.5, maximum displacement: 1 pixel.

After analysis, reconstructions were processed in Fiji/ImageJ using the similar method described to analyze SIM reconstructions. The edges of STORM clusters were set as 1/e the maximum intensity value instead of ½ the maximum intensity.

### Immunoblotting.

P10 retinas were collected in 2× protease inhibitor (Roche) diluted in 1× PBS (6.7 mM PO_4_ with Ca^2+^ and Mg^2+^) (Hyclone) from WT, *Rd16*, NN, and KO animals and sonicated in 1× SDS sample buffer (250 mM Tris pH 6.8, 10% SDS, 30% glycerol, 5% β-mercaptoethanol), and 1 retina per lane was loaded on a 3%–8% Tris-Acetate gel for SDS-PAGE. Gels were run in 1× Tris-Acetate SDS Running Buffer (20×: 50 mM Tricine, 50 mM Tris base, 0.1% SDS, pH 8.24; Bio-Rad). Proteins were transferred to unsupported nitrocellulose membranes in 1x Tris-Acetate transfer buffer (20×: 25 mM Bicine, 25 mM Bis-Tris free base, 1 mM EDTA, pH 7.2; Bio-Rad) overnight at room temperature. Membrane was blocked in 5% skim milk for 1 hour at room temperature. Primary antibodies were added and incubated overnight in 5% nonfat dry milk at 4°C. Secondary HRP antibodies (LI-COR Biosciences) were incubated with the membranes for 2 hours with shaking at room temperature. Membranes were imaged on an Azure Scanner with a 20- to 90-second exposure time. Image processing was performed in Abode Photoshop. All image contrast adjustments were applied identically to all images being compared.

### Statistics.

Numbers of animals and/or replicates are indicated in each figure legend. Two-way comparisons between antigens in 1 genotype were analyzed by Student’s 2-tailed *t* test. Multiple comparisons were analyzed by 1-way ANOVA with Tukey’s or Dunnett’s post hoc correction for multiple comparisons. *P* values less than 0.05 were considered statistically significant.

### Study approval.

All animal studies were approved by the Baylor College of Medicine Institutional Animal Care and Use Committee. They adhered to the ARVO guidelines for the humane treatment and ethical use of animals for vision research.

## Author contributions

VLP designed and executed experiments, analyzed data, and wrote the manuscript. She was assigned first authorship for initiating the project and writing the initial manuscript. ARM and MAR designed and executed experiments, analyzed data, wrote sections of the manuscript and helped edit it. TGW supervised the project, analyzed data, and helped edit the manuscript.

## Supplementary Material

Supplemental data

## Version 1 09/14/2021

In-Press Preview

## Version 2 10/22/2021

Electronic publication

## References

[1] Badano JL (2006). The ciliopathies: an emerging class of human genetic disorders. Annu Rev Genomics Hum Genet.

[2] Zariwala MA (2007). Genetic defects in ciliary structure and function. Annu Rev Physiol.

[3] Bujakowska KM (2017). Photoreceptor cilia and retinal ciliopathies. Cold Spring Harb Perspect Biol.

[4] Weleber RG et al., eds. *GeneReviews((R))*. University of Washington, Seattle; 1993.

[5] den Hollander AI (2006). Mutations in the CEP290 (NPHP6) gene are a frequent cause of Leber congenital amaurosis. Am J Hum Genet.

[6] den Hollander AI (2008). Leber congenital amaurosis: genes, proteins and disease mechanisms. Prog Retin Eye Res.

[7] Perrault I (2007). Spectrum of NPHP6/CEP290 mutations in Leber congenital amaurosis and delineation of the associated phenotype. Hum Mutat.

[8] Birtel J (2018). Next-generation sequencing identifies unexpected genotype-phenotype correlations in patients with retinitis pigmentosa. PLoS One.

[9] Valente EM (2006). Mutations in CEP290, which encodes a centrosomal protein, cause pleiotropic forms of Joubert syndrome. Nat Genet.

[10] Baala L (2007). Pleiotropic effects of CEP290 (NPHP6) mutations extend to Meckel syndrome. Am J Hum Genet.

[11] Brancati F (2007). CEP290 mutations are frequently identified in the oculo-renal form of Joubert syndrome-related disorders. Am J Hum Genet.

[12] Frank V (2008). Mutations of the CEP290 gene encoding a centrosomal protein cause Meckel-Gruber syndrome. Hum Mutat.

[13] Leitch CC (2008). Hypomorphic mutations in syndromic encephalocele genes are associated with Bardet-Biedl syndrome. Nat Genet.

[14] Coppieters F (2010). CEP290, a gene with many faces: mutation overview and presentation of CEP290base. Hum Mutat.

[15] Pazour GJ, Witman GB (2003). The vertebrate primary cilium is a sensory organelle. Curr Opin Cell Biol.

[16] Satir P, Christensen ST (2007). Overview of structure and function of mammalian cilia. Annu Rev Physiol.

[17] Singla V, Reiter JF (2006). The primary cilium as the cell’s antenna: signaling at a sensory organelle. Science.

[18] Yang TT (2015). Superresolution pattern recognition reveals the architectural map of the ciliary transition zone. Sci Rep.

[19] Ringo DL (1967). Flagellar motion and fine structure of the flagellar apparatus in Chlamydomonas. J Cell Biol.

[20] Gilula NB, Satir P (1972). The ciliary necklace. A ciliary membrane specialization. J Cell Biol.

[21] Sun S (2019). Three-dimensional architecture of epithelial primary cilia. Proc Natl Acad Sci U S A.

[22] Robichaux MA (2019). Defining the layers of a sensory cilium with STORM and cryoelectron nanoscopy. Proc Natl Acad Sci U S A.

[23] Pazour GJ (2002). Polycystin-2 localizes to kidney cilia and the ciliary level is elevated in orpk mice with polycystic kidney disease. Curr Biol.

[24] Handel M (1999). Selective targeting of somatostatin receptor 3 to neuronal cilia. Neuroscience.

[25] Wensel TG (2021). Structure and dynamics of photoreceptor sensory cilia. Pflugers Arch.

[26] Gilliam JC (2012). Three-dimensional architecture of the rod sensory cilium and its disruption in retinal neurodegeneration. Cell.

[27] Salisbury JL (1995). Centrin, centrosomes, and mitotic spindle poles. Curr Opin Cell Biol.

[28] Wolfrum U (1995). Centrin in the photoreceptor cells of mammalian retinae. Cell Motil Cytoskeleton.

[29] Wolfrum U, Salisbury JL (1998). Expression of centrin isoforms in the mammalian retina. Exp Cell Res.

[30] Laoukili J (2000). Differential expression and cellular distribution of centrin isoforms during human ciliated cell differentiation in vitro. J Cell Sci.

[31] Pulvermuller A (2002). Calcium-dependent assembly of centrin-G-protein complex in photoreceptor cells. Mol Cell Biol.

[32] Muresan V, Besharse JC (1994). Complex intermolecular interactions maintain a stable linkage between the photoreceptor connecting cilium axoneme and plasma membrane. Cell Motil Cytoskeleton.

[33] Kim J (2008). CEP290 interacts with the centriolar satellite component PCM-1 and is required for Rab8 localization to the primary cilium. Hum Mol Genet.

[34] Tsang WY (2008). CP110 suppresses primary cilia formation through its interaction with CEP290, a protein deficient in human ciliary disease. Dev Cell.

[35] Garcia-Gonzalo FR (2011). A transition zone complex regulates mammalian ciliogenesis and ciliary membrane composition. Nat Genet.

[36] Dharmat R (2018). SPATA7 maintains a novel photoreceptor-specific zone in the distal connecting cilium. J Cell Biol.

[37] Cox G, Sheppard CJ (2004). Practical limits of resolution in confocal and non-linear microscopy. Microsc Res Tech.

[38] Gustafsson MG (2000). Surpassing the lateral resolution limit by a factor of two using structured illumination microscopy. J Microsc.

[39] Kim D (2015). Correlative stochastic optical reconstruction microscopy and electron microscopy. PLoS One.

[40] Graser S (2007). Cep164, a novel centriole appendage protein required for primary cilium formation. J Cell Biol.

[41] Acu ID (2010). Coordination of centrosome homeostasis and DNA repair is intact in MCF-7 and disrupted in MDA-MB 231 breast cancer cells. Cancer Res.

[42] Piperno G, Fuller MT (1985). Monoclonal antibodies specific for an acetylated form of alpha-tubulin recognize the antigen in cilia and flagella from a variety of organisms. J Cell Biol.

[43] Molday RS, Molday LL (1979). Identification and characterization of multiple forms of rhodopsin and minor proteins in frog and bovine rod outer segment disc membranes. Electrophoresis, lectin labeling, and proteolysis studies. J Biol Chem.

[44] Yang TT (2018). Super-resolution architecture of mammalian centriole distal appendages reveals distinct blade and matrix functional components. Nat Commun.

[45] Craige B (2010). CEP290 tethers flagellar transition zone microtubules to the membrane and regulates flagellar protein content. J Cell Biol.

[46] Horst CJ (1987). Cytoskeletal-membrane interactions: a stable interaction between cell surface glycoconjugates and doublet microtubules of the photoreceptor connecting cilium. J Cell Biol.

[47] Tokuyasu K, Yamada E (1959). The fine structure of the retina studied with the electron microscope. IV. Morphogenesis of outer segments of retinal rods. J Biophys Biochem Cytol.

[48] Rohlich P (1975). The sensory cilium of retinal rods is analogous to the transitional zone of motile cilia. Cell Tissue Res.

[49] Dawe HR (2007). The Meckel-Gruber Syndrome proteins MKS1 and meckelin interact and are required for primary cilium formation. Hum Mol Genet.

[50] Tiwari S (2013). Meckelin 3 is necessary for photoreceptor outer segment development in rat Meckel syndrome. PLoS One.

[51] Williams CL (2011). MKS and NPHP modules cooperate to establish basal body/transition zone membrane associations and ciliary gate function during ciliogenesis. J Cell Biol.

[52] Gerhardt C (2015). The transition zone protein Rpgrip1l regulates proteasomal activity at the primary cilium. J Cell Biol.

[53] Hong DH (2000). A retinitis pigmentosa GTPase regulator (RPGR)-deficient mouse model for X-linked retinitis pigmentosa (RP3). Proc Natl Acad Sci U S A.

[54] Chang B (2016). Mouse models as tools to identify genetic pathways for retinal degeneration, as exemplified by Leber’s congenital amaurosis. Methods Mol Biol.

[55] Cideciyan AV (2011). Cone photoreceptors are the main targets for gene therapy of NPHP5 (IQCB1) or NPHP6 (CEP290) blindness: generation of an all-cone Nphp6 hypomorph mouse that mimics the human retinal ciliopathy. Hum Mol Genet.

[56] Chang B (2006). In-frame deletion in a novel centrosomal/ciliary protein CEP290/NPHP6 perturbs its interaction with RPGR and results in early-onset retinal degeneration in the rd16 mouse. Hum Mol Genet.

[57] Lancaster MA (2011). Defective Wnt-dependent cerebellar midline fusion in a mouse model of Joubert syndrome. Nat Med.

[58] Hynes AM (2014). Murine Joubert syndrome reveals Hedgehog signaling defects as a potential therapeutic target for nephronophthisis. Proc Natl Acad Sci U S A.

[59] Datta P (2019). The myosin-tail homology domain of centrosomal protein 290 is essential for protein confinement between the inner and outer segments in photoreceptors. J Biol Chem.

[60] Rachel RA (2015). CEP290 alleles in mice disrupt tissue-specific cilia biogenesis and recapitulate features of syndromic ciliopathies. Hum Mol Genet.

[61] Drivas TG (2013). Disruption of CEP290 microtubule/membrane-binding domains causes retinal degeneration. J Clin Invest.

[62] Jana SC (2018). Differential regulation of transition zone and centriole proteins contributes to ciliary base diversity. Nat Cell Biol.

[63] Stone EM (2011). Variations in NPHP5 in patients with nonsyndromic leber congenital amaurosis and Senior-Loken syndrome. Arch Ophthalmol.

[64] Otto EA (2005). Nephrocystin-5, a ciliary IQ domain protein, is mutated in Senior-Loken syndrome and interacts with RPGR and calmodulin. Nat Genet.

[65] Estrada-Cuzcano A (2011). IQCB1 mutations in patients with leber congenital amaurosis. Invest Ophthalmol Vis Sci.

[66] Barbelanne M (2013). Pathogenic NPHP5 mutations impair protein interaction with Cep290, a prerequisite for ciliogenesis. Hum Mol Genet.

[67] Barbelanne M (2015). Nephrocystin proteins NPHP5 and Cep290 regulate BBSome integrity, ciliary trafficking and cargo delivery. Hum Mol Genet.

[68] Ronquillo CC (2016). Ciliopathy-associated IQCB1/NPHP5 protein is required for mouse photoreceptor outer segment formation. FASEB J.

[69] Basinger S (1976). Rhodopsin in the rod outer segment plasma membrane. J Cell Biol.

[70] Heitzmann H (1972). Rhodopsin is the predominant protein of rod outer segment membranes. Nat New Biol.

[71] Awata J (2014). NPHP4 controls ciliary trafficking of membrane proteins and large soluble proteins at the transition zone. J Cell Sci.

[72] Gogendeau D (2020). MKS-NPHP module proteins control ciliary shedding at the transition zone. PLoS Biol.

[73] Megaw RD (2015). RPGR: its role in photoreceptor physiology, human disease, and future therapies. Exp Eye Res.

[74] Li T (2014). Leber congenital amaurosis caused by mutations in RPGRIP1. Cold Spring Harb Perspect Med.

[75] Shu X (2005). RPGR ORF15 isoform co-localizes with RPGRIP1 at centrioles and basal bodies and interacts with nucleophosmin. Hum Mol Genet.

[76] Skiba NP (2021). TMEM67, TMEM237, and embigin in complex with monocarboxylate transporter MCT1 are unique components of the photoreceptor outer segment plasma membrane. Mol Cell Proteomics.

[77] Lau L (2012). STED microscopy with optimized labeling density reveals 9-fold arrangement of a centriole protein. Biophys J.

[78] Tillberg PW (2016). Protein-retention expansion microscopy of cells and tissues labeled using standard fluorescent proteins and antibodies. Nat Biotechnol.

[79] Chang JB (2017). Iterative expansion microscopy. Nat Methods.

[80] Macaluso FP (2016). CLEM methods for studying primary cilia. Methods Mol Biol.

[81] Norris RP (2017). Localization of phosphorylated connexin 43 using serial section immunogold electron microscopy. J Cell Sci.

[82] Lopes CA (2011). Centriolar satellites are assembly points for proteins implicated in human ciliopathies, including oral-facial-digital syndrome 1. J Cell Sci.

[83] Stowe TR (2012). The centriolar satellite proteins Cep72 and Cep290 interact and are required for recruitment of BBS proteins to the cilium. Mol Biol Cell.

