# Supplementary material for: Super-resolution microscopy reveals photoreceptor-specific subciliary location and function of ciliopathy-associated protein CEP290
Source: JCI Insight. 2021 Oct 22;6(20):e145256. doi: 10.1172/jci.insight.145256 (PMC8564900; doi:10.1172/jci.insight.145256)
Supplement: Supplemental data [file jciinsight-6-145256-s160.pdf]

Supplemental Methods

Genotyping:

Rd16 animals were identified by mutagenically separated PCR using the following primers – mutant forward 5'-CCACCCCATCTTCATGTG-3', wild type forward 5'-TGTGAAGTGAACCCATGAATAG-3', and universal reverse 5'-CCCTCCAATATCAGGAAATGA-3'. *Cep290*$^{tm1.1Jgg}$ animals were identified by mutagenically separated PCR using the following primers – mutant forward 5'-TGGAAGACCAGGCTTCAGAG-3', mutant reverse 5'-GGCTCACTGTGATCTTGTGC-3', wild type forward 5'-GTAAGTGCCCGACAGCTACC-3', and wild type reverse 5'-AGCGCAGTGCAGAGTATGTG-3'. All mouse strains were screened for the absence of *PDE6b*^{rd1} and *Crb1*^{rd8} alleles.

Antibodies.

The following commercial antibodies were used: CEP290 (C-terminus), Bethyl Laboratory, A301-659A; acetylated α-tubulin, Santa Cruz, sc-23950; NPHP5, Proteintech, 15747-1-AP; Centrin, pan-centrin antibody from EMD Millipore, 04-1624, raised against *Chlamydomonas* centrin and reported to recognize all mouse centrin isoforms 1-4 (41, 84); CEP164, Proteintech, 22227-1-AP; CEP164, Santa Cruz, sc-515403; MKS3 (cultured cells) Proteintech, 13975-1-AP; MKS3/TMEM67 (retina), ABclonal, A15549; CEP290/NPHP6 (N-terminus), BiCell, 90006; NPHP8/RPGRIP1L (retina), BiCell, 90008; RPGRIP1L (cultured cells), Proteintech, 55160-1-AP; wheat germ agglutinin (WGA)-A647, Molecular Probes, W32466; Atto488-Tuba1, Antibodies-online.com, ABIN1169085. Rat anti-CEP290 antibody was a gift from the Swaroop laboratory. Mouse anti-1D4 was generated in house. For SIM and confocal imaging, antibodies were used at a concentration of 1 µg/150 µL. For STORM, antibodies were used at a concentration of 10 µg/mL, with the exception of rat anti-CEP290 antibody and Atto488-Tuba1 which were used at concentrations of 5 µg/mL and 7.5 µg/mL, respectively.

Sample preparation for confocal, deconvolution, and SIM immunofluorescence microscopy

Eye cups from mice aged 4-8 months (Figures 2-5) or 10 days postnatal (Figures 6, 7, 9) were fixed for five minutes in 1% PFA (Electron Microscopy Science) diluted in 1x PBS, cryopreserved in 30% sucrose overnight at 4C, embedded in Optimal Cutting Temperature (OCT) media, and flash frozen by liquid nitrogen. Eye cups were cryosectioned at 8 μm thickness. Sections were blocked in 2% Normal Goat Serum (NGS, Fitzgerald Industries) or Normal Donkey Serum (NDS, Jackson ImmunoResearch) + 2% Fish Scale Gelatin (Sigma) + 2% Bovine Serum Albumin (Sigma) + 0.2% Triton X-100 dilute in 1x PBS and probed with 0.5 - 1 μg of primary antibody at room temperature overnight. After goat or donkey secondary antibody labeling (Thermo Fisher) (1:500), sections were mounted with Vectashield Antifade (Vector Labs) or Prolong Glass (Thermo Scientific) and covered with 1.5 coverslip (Leica) for imaging on a Leica TCS-SP5 confocal microscope. Prolong Glass mounting media was selected because the refractive index (1.520) is more compatible with SIM and reduced chromatic artifacts.

STORM immunohistochemistry and resin embedding

Retinas from 6-8 week old WT mice were immunolabeled for STORM using a protocol we developed previously (Robichaux et al 2019). Whole retinas were stained in a solution following a two-step protocol. First, retinas were dissected unfixed in ice cold Ames' media (Sigma) and immediately blocked in 10% NGS (Fitzgerald Industries) + 0.2% saponin (Sigma) + 1x Protease Inhibitor Cocktail (GenDepot) diluted in 1x Ames' media for 2 hours at 4°C. Primary antibodies (5-10 μg each) were added to the blocking

buffer and incubated at 4°C for 20-22 hours. Retinas were washed 3 times for 5 minutes in 2% NGS in Ames' media on ice before secondary antibodies were added to the same buffer and incubated at 4°C for 2 hours. Secondary antibodies used (8 µg each): F(ab')2-goat anti-mouse IgG Alexa 647 & F(ab')2-goat anti-rabbit IgG Alexa 555 (Thermo Fisher). In WGA-Alexa647 labeling experiments, F(ab')2-goat anti-rabbit IgG Alexa 555 (Thermo Fisher) was used for dual labeling. Retinas were washed in 2% NGS/Ames 6 times for 5 minutes each on ice and fixed in 4% paraformaldehyde diluted in 1xPBS for 15 minutes at room temperature.

Next, retinas were re-blocked in 10% normal goat serum + 0.2% saponin diluted in 1xPBS for 2 hours at room temperature. Primary antibodies (5-10 µg each) were re-added to the blocking buffer and incubated for 2 days at 4°C. After this incubation, retinas were washed 4x for 10 minutes each in 2% NGS/1x PBS. The same secondary antibodies were added to the wash buffer as before (8 µg each) for overnight incubation at 4°C. Retinas were washed 6x in 2% NGS/1x PBS for 5 minutes each before post-fixation in 3% formaldehyde diluted in 1x PBS for 1 hour at room temperature.

Post-fixed retinas were dehydrated in a series of ethanol washes (15 minutes each: 50%, 70%, 90%, 100%, 100%) followed by embedding steps of increasing concentrations with Ultra Bed Epoxy Resin (Electron Microscopy Sciences) to ethanol (2 hours each: 25%:75%, 50%:50%, 75%:25%, 100% resin twice). Embedded retinas were cured on the top shelf of a 65'C baking oven for 20 hours. 500 nm - 1 µm sections were cut on a UCT or UC6 Leica Ultramicrotome and dried directly onto glass-bottom dishes (MatTek 35 mm dish, No. 1.5 coverslip).

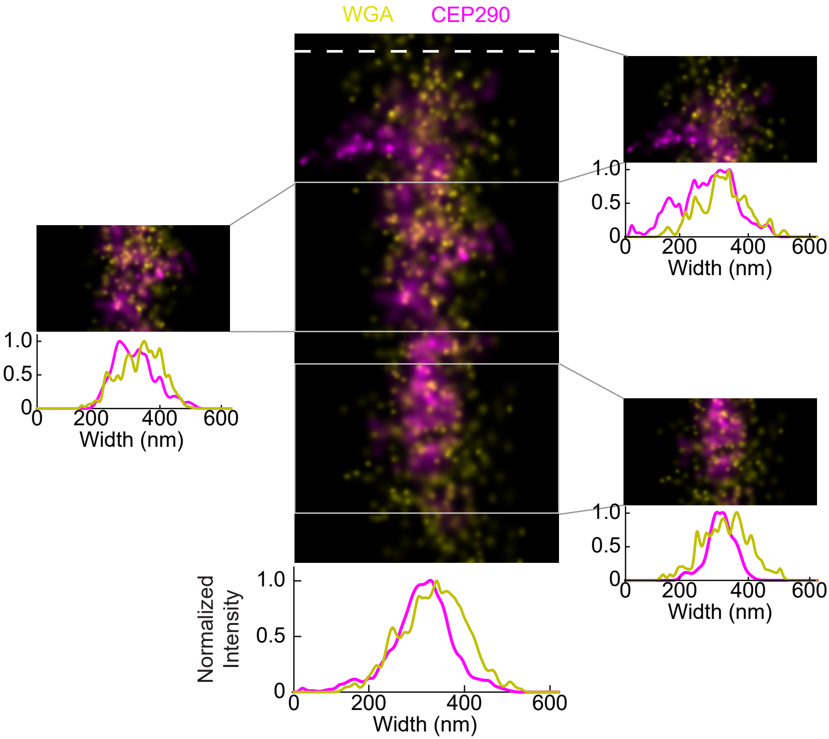

Supplemental Figure 1. Variation in the radial extent of CEP290 and WGA localization along the CC axis visualized by STORM. Larger image and intensity plot is from Figure 2F. Each highlighted region showcases 330nm in length along the original image and is accompanied by a row average intensity plots. The highlighted regions and their plots demonstrate the changing nature of CEP290 and WGA in relationship to one another throughout the cilium. Dimensions of the larger image are 1200nm x 600nm, while the highlighted regions are 330nm x 600nm. WGA, wheat germ agglutinin. Dotted line indicates CC/OS border.

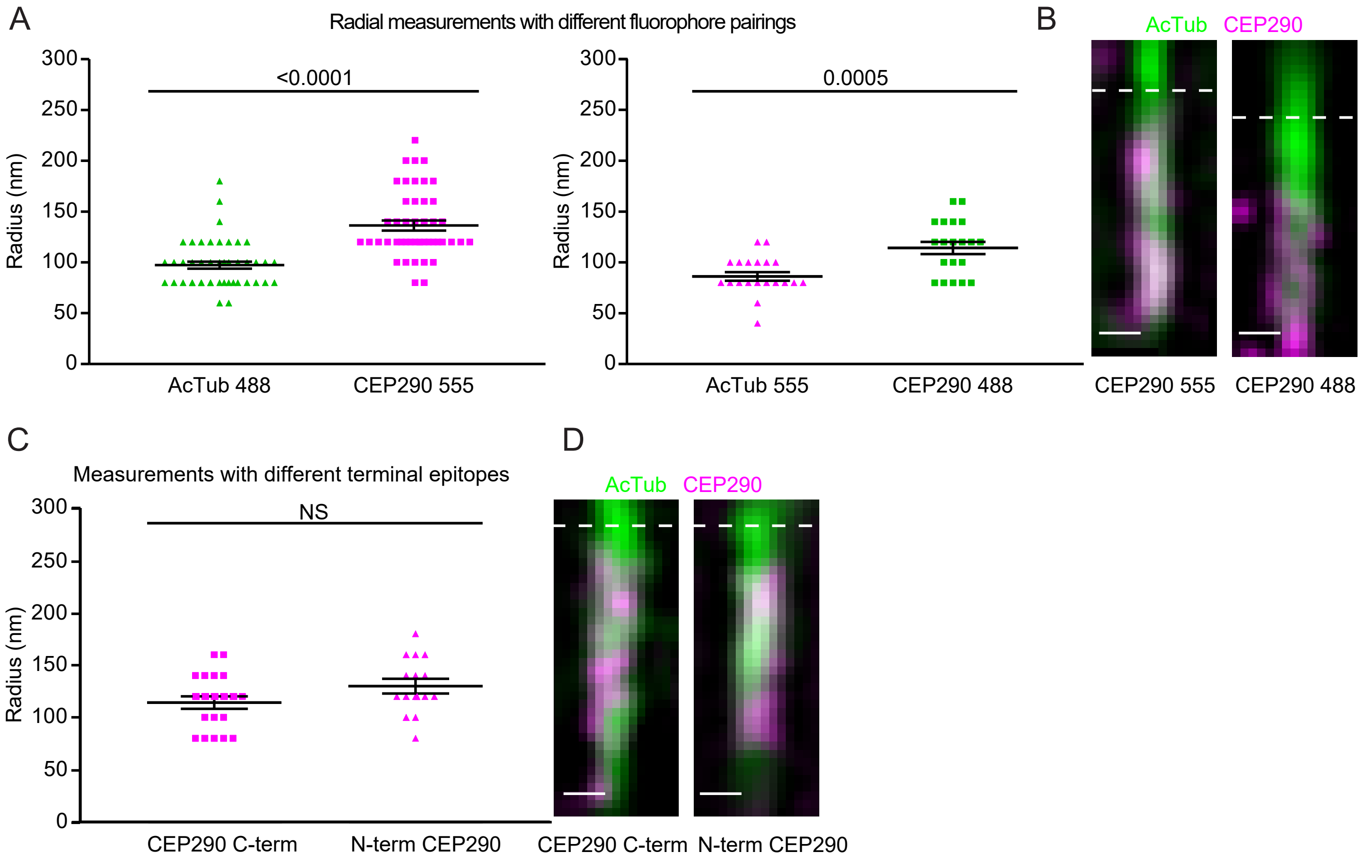

Supplemental Figure 2. Comparisons of signals using swapped reagents. (A) Dot plots with averages and standard deviations of radial measurements with swapped fluorophores. (B) SIM images of representative cilia from each fluorophore pairing. (C) Dot plot with averages and standard deviations of radial measurements with C- or N-terminal specific CEP290 antibodies. (D) SIM images of representative cilia labelled with antibodies identifying different CEP290 epitopes. 33% of the maximum intensity value of each channel was used to determine the boundary for the measurement of each cilium. Measurements were compared with Student's t-test with Welch correction. Dotted line indicates CC/OS border. Scale bar 200nm. AcTub, acetylated -tubulin.

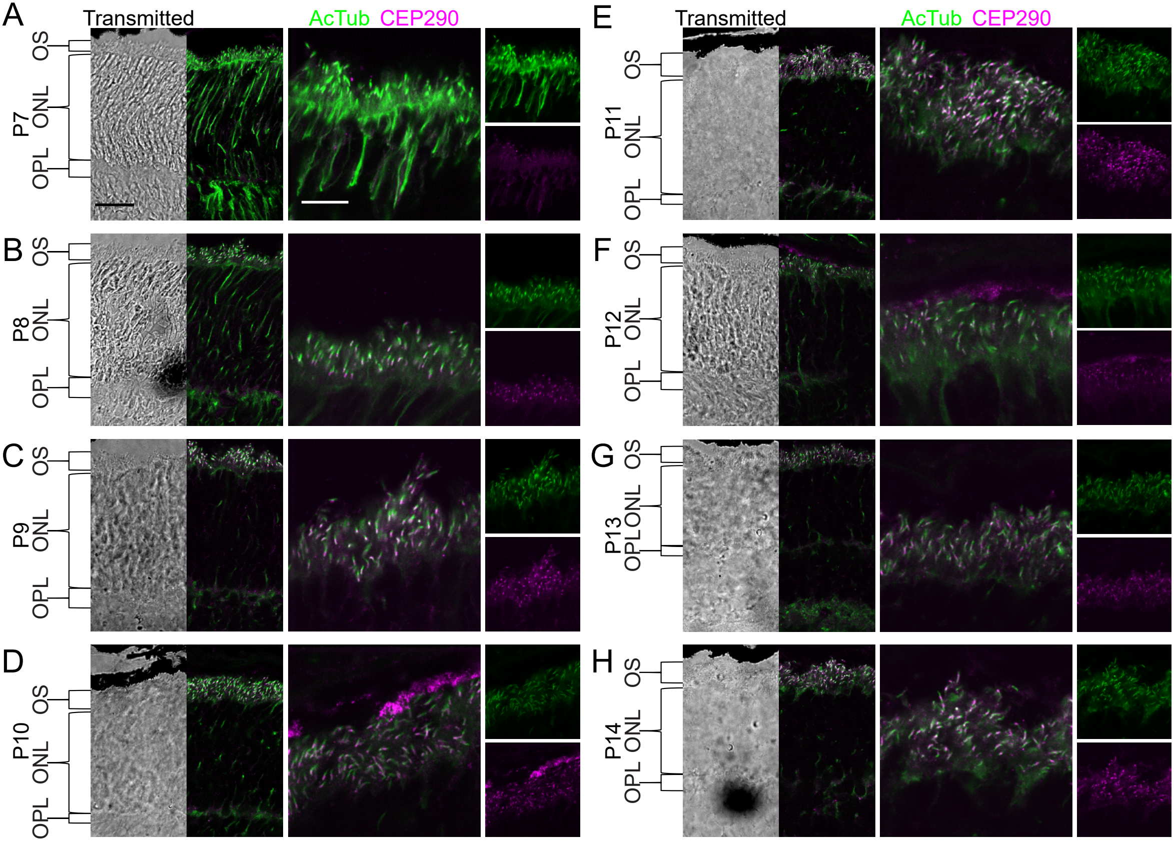

Supplemental Figure 3. Time course of CEP290 localization in rd16 mutant. (A-H) Confocal images of retina from rd16 animals ages P7 (A), P8 (B), P9 (C), P10 (D), P11 (E), P12 (F), P13 (G), and P14 (H). Low magnification image (left) shows transmitted image and AcTub and CEP290 labelled retina. Scale bar 25 μm. Zoomed images (right) highlights AcTub and CEP290 labelled cilia with separate channels. Scale bar 10 μm. Retinas were imaged from littermates. Same acquisition and processing settings were applied to all data. AcTub, acetylated α-tubulin; OS, outer segment; ONL, outer nuclear layer; OPL, outer plexiform layer; rd16, *Cep290[rd16]*.

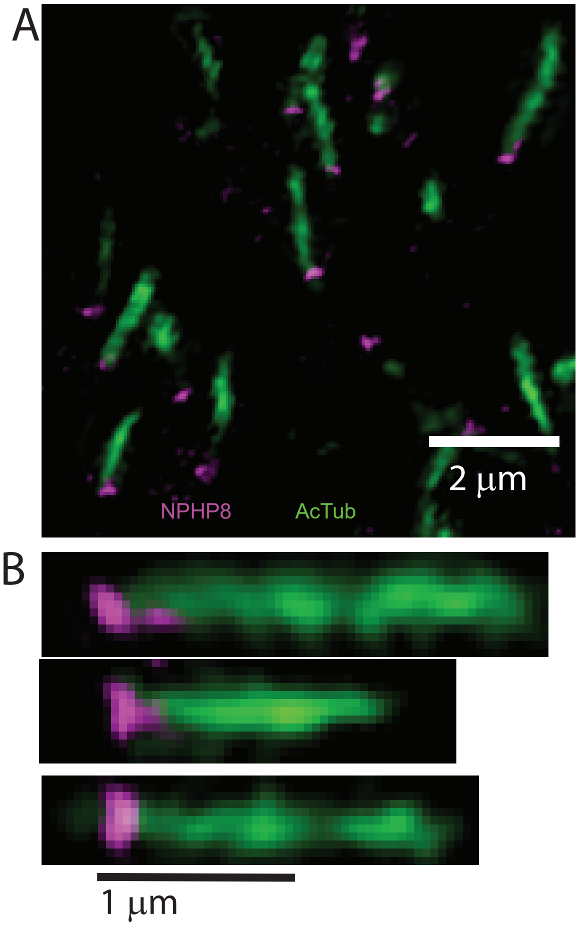

Supplementary Figure 4. Localization of NPHP8 in photoreceptor cilia. SIM images at low (A) and high (B) magnifications showing staining for NPHP8 and acetylated α tubulin (Actub).

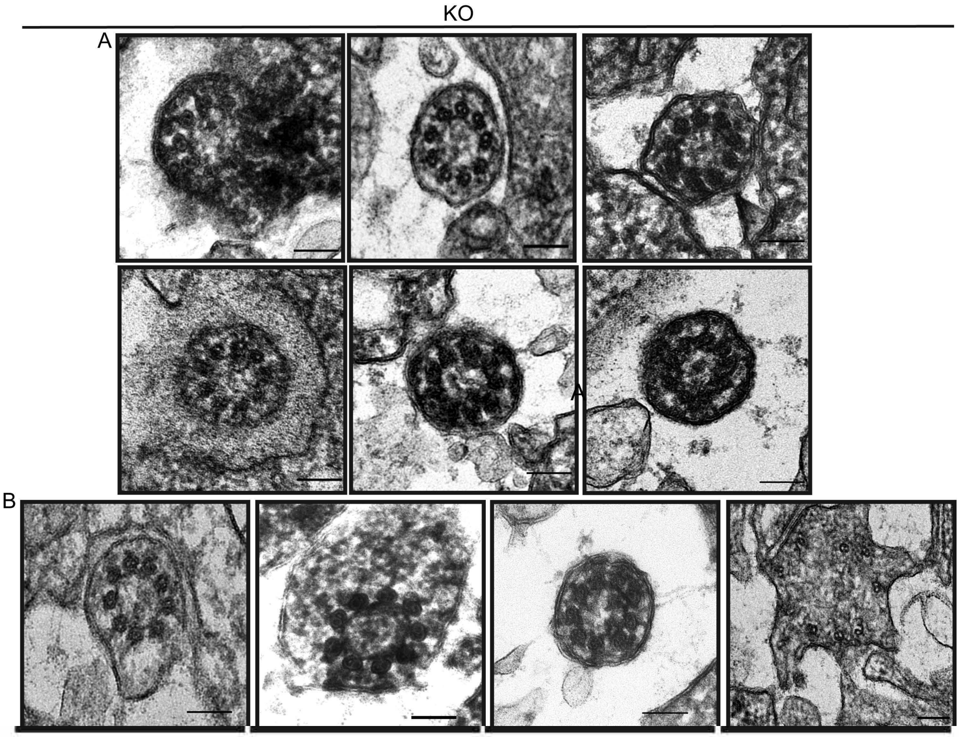

Supplemental Figure 5. TEM images of cross-sections of CC in KO retina at P10. (A) Upper two rows: Examples of CC with Y-links. (B) Bottom row: Examples of aberrant CC cross sections frequently seen in KO near distal end of CC. Scale bar = 100nm.

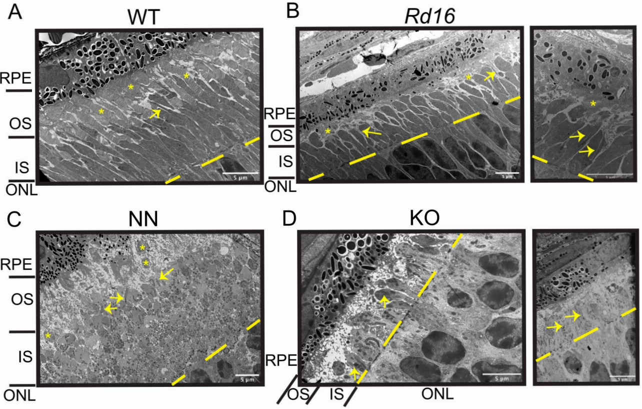

Supplemental Figure 6. Low magnification Cep290 mutant transition electron microscopy. Uncropped, low magnification images from P10 WT (A), rd16 (B), NN(C), and KO(D) animals; higher magnification images are presented in Figure 9. Scale bar 5 m. Same acquisition and processing settings were applied to all data. *Asterisks - OS discs, arrow – CC, dashed lines – outer limiting membrane (IS/ONL interface). RPE, retinal pigment epithelial; OS, outer segment; IS, inner segment; ONL, outer nuclear layer; WT, wild type; rd16, Cep290rd16; NN, near null-Cep290tm1.1Jgg; KO, knockout-Cep290.

Uncropped Western Blots

For Figure 6 of Potter et al. JCI Insight paper, “**Superresolution microscopy reveals photoreceptor-specific subciliary location and function of ciliopathy-associated protein, Cep290**”

P10 retina

Cep290 (n-term)

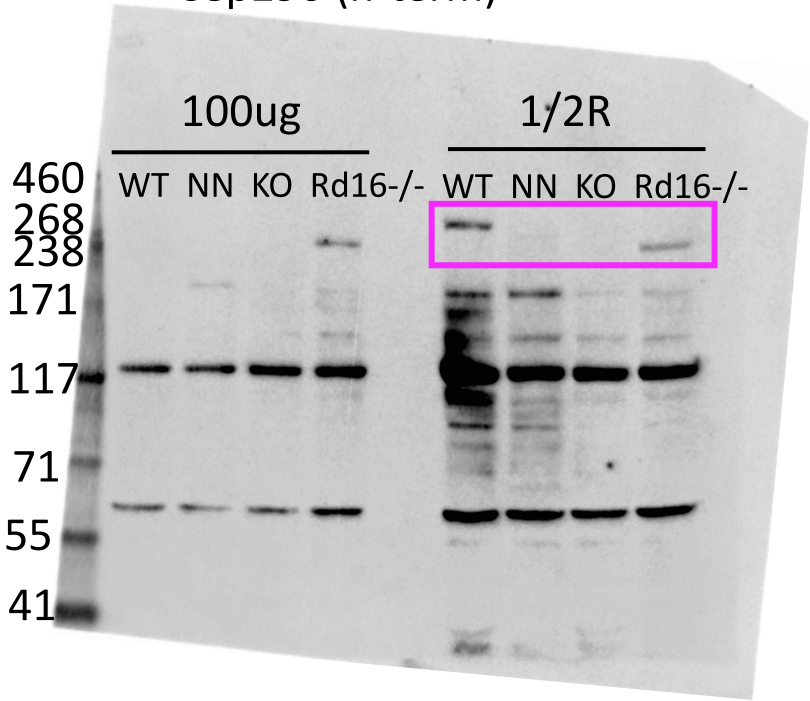

Cep290 (c-term)

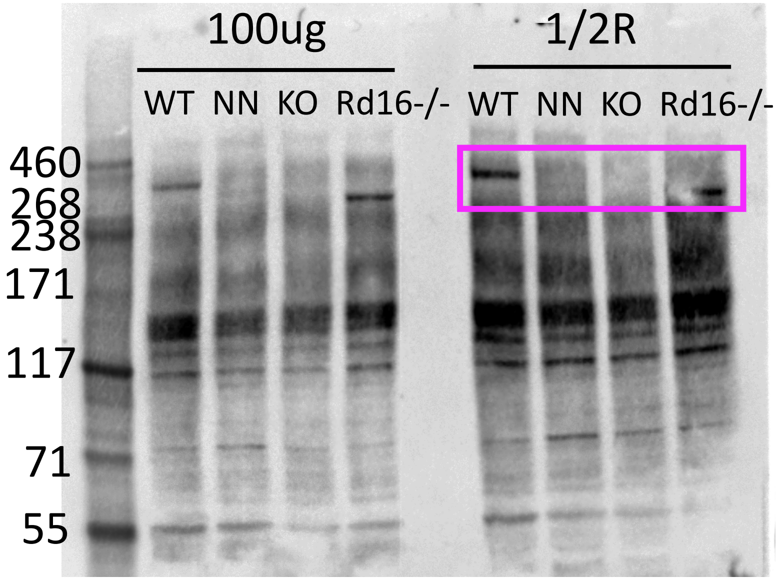

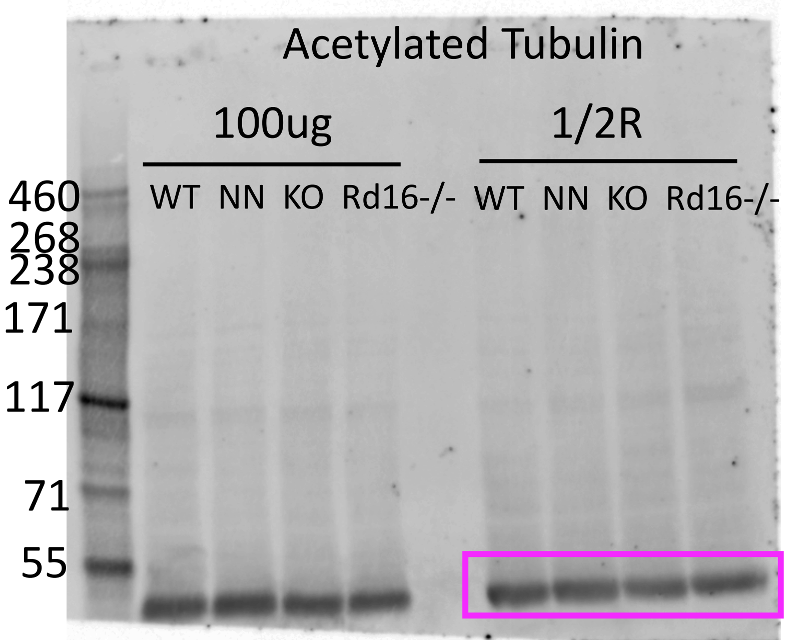

Same gel as above, using IR800 for acetylated tubulin after being stained and imaged for cep290c

The indicated lanes were used for Figure 6K.
The same samples were used for both gels. They were prepared and run at the same time; The CEP290 N-terminal antibody was used on one blot (left), and the C-terminal antibody on the other (upper right). The gel used for the CEP290 C-terminal antibody was also probed by an antibody for acetylated alpha tubulin (lower right) using a different secondary with a different wavelength IR dye as loading control for both gels.

Tris-Acetate Gels, 20V O/N transfer
